# Monocyte LOXHD1 and RHOB Expression Predictive of Progressive Systemic Sclerosis–Associated Interstitial Lung Disease

**DOI:** 10.1002/acr.25619

**Published:** 2026-01-29

**Authors:** Cristina M. Padilla, Robert Lafyatis, Kevin F. Gibson, Christina Morse, Eleanor Valenzi, Xiaoping Chen, Xiaoyun Li, Yanwu Zhao, Yongseok Park, Dana Ascherman, Jonathan Minden, Robyn Domsic, Yingze Zhang, Daniel J. Kass

**Affiliations:** ^1^ University of Pittsburgh Pittsburgh Pennsylvania; ^2^ Carnegie Mellon University Pittsburgh Pennsylvania

## Abstract

**Objective:**

A leading cause of death among patients with scleroderma (SSc), interstitial lung disease (ILD) remains challenging to prognosticate. The discovery of biomarkers that accurately determine which patients would benefit from close monitoring and aggressive therapy would be an essential clinical tool. We aimed to identify gene signatures that would predict progressive ILD.

**Methods:**

We compared previously identified serum biomarkers, bulk peripheral blood mononuclear cell (PBMC) RNA gene expression (39 patients with progressive SSc‐ILD and 43 patients with stable SSc‐ILD), and single‐cell RNA gene expression of PBMC (13 patients with progressive SSc‐ILD, 14 patients with stable SSc‐ILD, and 6 control participants).

**Results:**

In previous studies, male sex, Krebs von den Lungen 6 levels, and C‐reactive protein levels were predictors of progressive disease in patients. Monocyte expression of lipoxygenase homology domains 1 (LOXHD1) and Ras homolog gene family, member B (RHOB) strongly predicted progression, suggesting a key role in immune dysregulation. *LOXHD1* and related genes were enriched in inflammatory gene networks, which may support monocyte‐associated inflammation in patients. RHOB was consistently up‐regulated in both CD14+ and CD16+ monocytes across single‐cell and bulk RNA data, underscoring robust association with progressive disease. In contrast, ataxin‐2‐like (ATXN2L) protein was down‐regulated in patients with progressive disease, suggesting dysregulation of cellular stress responses. Additionally, S100 calcium‐binding protein A12 (S100A12), chitinase‐3‐like protein 1 (CHI3L1), and matrix metalloproteinase‐9 (MMP9), previously linked to tissue remodeling, were again associated with progression in bulk RNA sequencing and previous microarray studies, reinforcing their role in fibrosis.

**Conclusion:**

With several potential biomarkers of progressive disease in patients, the next step includes validation in larger, multicenter cohorts for use in clinical decision‐making.

## INTRODUCTION

Interstitial lung disease (ILD) in patients with systemic sclerosis (SSc) is the most lethal complication, occurring in patients with both limited cutaneous and diffuse cutaneous disease. The course of SSc‐ILD is quite variable. In some patients, the disease is mild and not progressive. Patients with mild disease on presentation have a better prognosis. On the other hand, patients with anti‐Scl‐70 autoantibodies tend to have more severe, progressive disease.

**SIGNIFICANCE & INNOVATIONS**

- Up‐regulated expression of lipoxygenase homology domains 1 and Ras homolog gene family, member B and down‐regulated expression of ataxin‐2‐like protein in monocytes are associated with progressive systemic sclerosis (SSc) interstitial lung disease (ILD) in patients and could be prognostic biomarkers.
- S100 calcium‐binding protein A12, chitinase‐3‐like protein 1, and matrix metalloproteinase‐9 are part of a cluster associated with progressive SSc‐ILD in patients and could represent true biomarkers of SSc‐ILD.
- Biomarkers that track using disease activity or prognostication of SSc‐ILD in patients are invaluable clinical and research tools, which could lead to improved outcomes and effective therapies.

Several serum biomarkers of disease severity have been explored. C‐reactive protein (CRP) is the most widely available and best studied prognostic biomarker. We have recently reviewed other biomarkers: surfactant protein‐D (SP‐D), Krebs von den Lungen 6 (KL‐6), osteopontin (SPP1), and C‐C motif chemokine 18 (CCL18), which have also been studied as serum biomarkers; however, none are strongly predictive of a decline in pulmonary function.^1^ Thus, the discovery of prognostic biomarkers of SSc‐ILD would provide a significant new tool for clinicians to decide which patients to treat aggressively and for clinical trialists to help stratify patients for inclusion into clinical trials.

Multiple lines of evidence support the importance of autoimmunity and inflammation in patients with SSc and SSc‐ILD. We have shown previously that patients with SSc‐ILD have profound changes in the inflammatory cell populations in the lungs.^2^ This includes marked alterations in gene expression by three different monocyte‐macrophage populations. Alveolar macrophages are marked by *FABP4*, *INHBA*, and *SERPING1*, and monocyte‐like macrophages marked by *CD14*, *FCN1*, *IL1B*, and *IL1R2*.^2^ A third population of macrophages (SPP1 macrophages) show markedly up‐regulated expression of *SPP1*, *MMP9*, and *CCL18* in patients with SSc‐ILD as well as in patients with idiopathic pulmonary fibrosis (IPF).^2^, ^3^, ^4^ In view of the observation that bleomycin‐induced fibrosis in SPP1‐deleted mice is attenuated and a similar macrophage phenotype is seen in other fibrotic diseases, these macrophages appear profibrotic.^5^ T and natural killer (NK) cell populations in patients with SSc‐ILD also show altered gene expression compared with lungs from healthy controls (HC).^6^

Because lung biopsies and bronchoalveolar lavage are not routinely required diagnostically in patients with SSc‐ILD, gene expression by peripheral blood mononuclear cells (PBMCs) provides a potential surrogate for assessing immune cell function and for discovery of prognostic biomarkers in patients with SSc‐ILD. Analyzing PBMC gene expression by microarray, we previously reported that some PBMCs from patients with SSc show an interferon (IFN) signature.^7^, ^8^ We have also previously identified genes up‐regulated in PBMCs from patients with SSc with new onset isolated pulmonary arterial hypertension (*CCR1*, *IL13RA1*, *TIMP2*, *AIF1*, *ICAM1*, and *IFNGR1*).^8^ Here, we compare previously identified serum biomarkers, bulk PBMC RNA gene expression, and single‐cell RNA gene expression in patients with progressive SSc‐ILD versus patients with stable SSc‐ILD. These studies highlight several gene expression prognostic biomarkers, particularly monocyte expression of *LOXHD1* and *RHOB*, for patients with progressive SSc‐ILD.

## PATIENTS AND METHODS

### Study cohort

Blood samples were obtained from patients with SSc in the Dorothy P and Richard P Simmons Center for Interstitial Lung Disease at the University of Pittsburgh Medical Center (UPMC; the Simmons Center) or the Falk Rheumatology Clinic, who completed baseline evaluations and consented to experimental studies using their venous phlebotomy samples. Complete descriptions of recruitment and clinical evaluations were previously detailed.^9^, ^10^ Spirometry and diffusing capacity were performed using American Thoracic Society standards and standard reference equations.^11^, ^12^, ^13^ This study was approved by the Institutional Review Board for Human Subject Research at the University of Pittsburgh (STUDY20030223). All participants provided written informed consent (in accordance with the Declaration of Helsinki) before their participation in this study.

**Figure 1:**
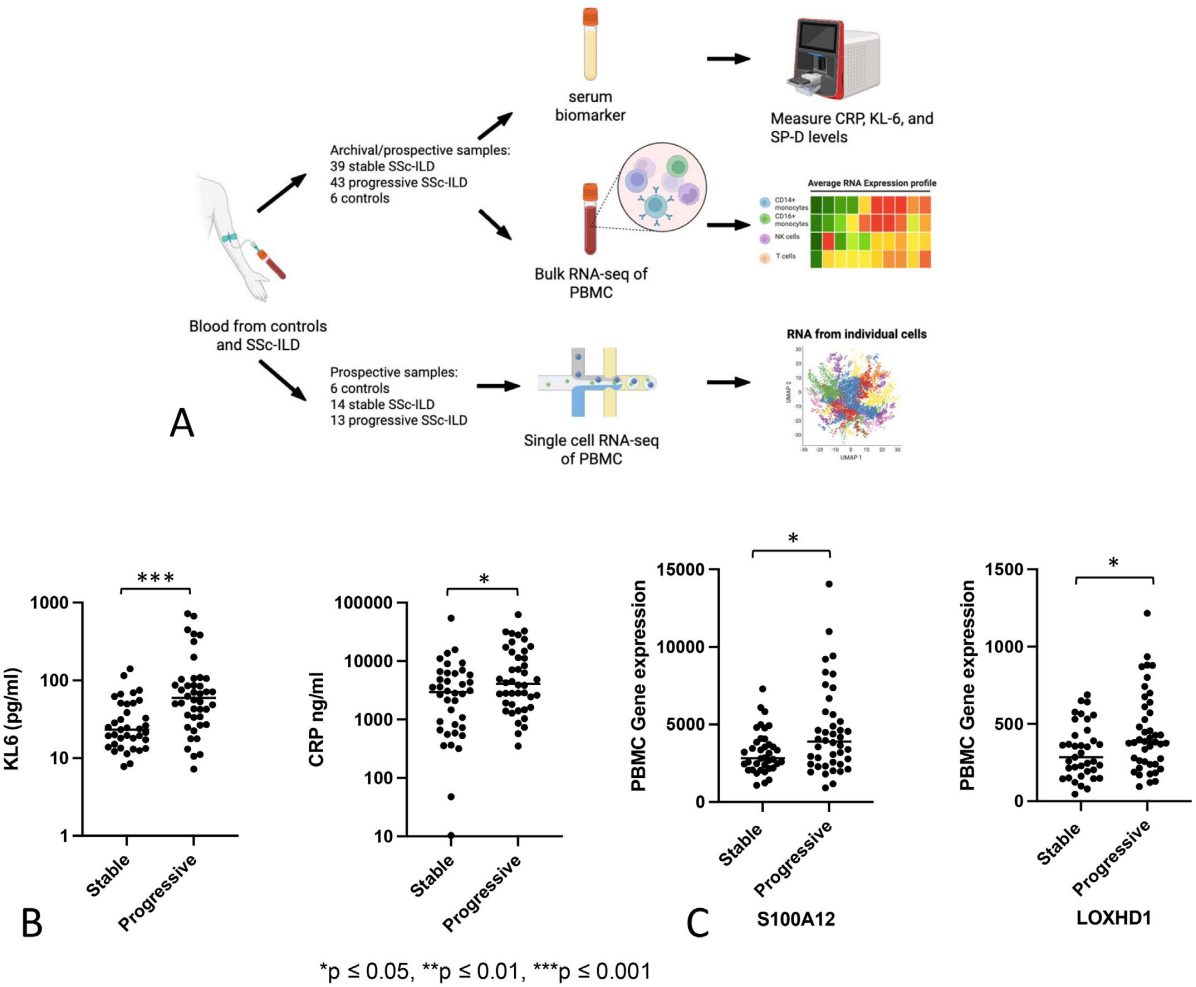
(A) Schematic overview of sample processing and data generation. (B) Bar plots comparing the serum levels of KL‐6 (*P* = 0.0004) and CRP (*P* = 0.03) between patients with stable SSc‐ILD and patients with progressive SSc‐ILD using two‐sided Mann‐Whitney. The horizontal line represents the average gene expression. (C) Bar plots exhibiting scRNA‐seq gene expression of monocyte‐like myeloid cells from patients with stable SSc‐ILD and progressive SSc‐ILD. These genes were found to have increased gene expression compared with healthy control (Supplementary Figure 1). *P ≤ 0.05. **P ≤ 0.01. ***P ≤ 0.001. CRP, C‐reactive protein; KL‐6, Krebs von den Lungen 6; NK, natural killer; PBMC, peripheral blood mononuclear cells; scRNA‐seq, single‐cell RNA sequencing; SP‐D, surfactant protein‐D; SSc‐ILD, scleroderma with interstitial lung disease. Color figure can be viewed in the online issue, which is available at http://onlinelibrary.wiley.com/doi/10.1002/acr.25619/abstract.

Archival PBMC samples and matched to date sera from the Simmons Center, and prospectively collected blood samples from patients seen in the clinic were studied. Samples collected at baseline (enrollment blood collection) from patients with SSc‐ILD were stratified into progressive disease or stable disease based on one of five criteria: (1) death within two years of enrollment from any cause; (2) lung transplant within two years after enrollment; (3) forced vital capacity (FVC) less than 50% at time of or within two years after enrollment; (4) greater than 10% decline in FVC over two years comparing both before and after blood collection; or (5) initiated on new immunosuppressive medication for ILD within three months of blood collection. Patients not meeting one of these criteria were considered to have stable disease at the time of enrollment. Patients for whom criteria were ambiguous were adjudicated as stable or progressive by chart review by Drs. Lafyatis and Kass. Demographic information and autoantibody reactivities were recorded for each patient when available from the medical chart. Figure 1A shows how both prospective and archival samples were used.

### Serum biomarker analyses

CRP, KL‐6, and SP‐D levels were analyzed using citrate plasma collected at the same time as PBMC from each patient. CRP and SP‐D were analyzed using Quantikine enzyme‐linked immunosorbent assay kits (DCRP00 and DSFPD0, R&D Systems). KL‐6 levels were analyzed using Luminex kit (LXSAHM‐01, R&D Systems) and Bioplex 200 (Bio‐Rad Labs). Sera collected at the same time as PBMC from each participant were used for analyzing creatine phosphokinase (CPK) levels through the UPMC central laboratory using an International Federation of Clinical Chemistry and Laboratory Medicine – creatine kinase (*N*‐acetylcysteine) kit on Beckman Coulter AU680 and AU5800 analyzers. The two‐sided Mann‐Whitney U test determined statistical significance.

### RNA isolation

For the historical samples, PBMCs were isolated using citrate cell preparation tube (CPT) vacutainer tubes (Beckman‐Dickerson) and stored in either Trizol or RNAlater (Thermo‐Fisher) at −80°C until RNA isolation. Samples stored in RNAlater were lysed in Trizol during RNA isolation procedure. RNA isolation was followed standard protocol using Qiagen Rneasy mini kit (Qiagen). All RNA samples were quantified using Agilent RNA 6000 Nano Kit and Bioanalyzer (Agilent 2100). All samples passed quality control with an integrity number greater than eight.

### RNA sequencing library generation and sequencing

RNA was assessed for quality (Agilent TapeStation 4150) and RNA concentration quantified (Qubit FLEX fluorometer). Libraries were generated with the Illumina Stranded messenger RNA Library Prep kit (catalog #20040534) according to the manufacturer's instructions. Briefly, 100 ng of input RNA was used for each sample. Following adapter ligation, 13 cycles of indexing PCR were completed, using Integrated DNA Technologies (IDT) for Illumina RNA UD Indexes (Illumina, catalog #20040553‐6). Libraries were quality and quantity and assessed (Qubit FLEX fluorometer and Agilent TapeStation 4150). Libraries were normalized and pooled to 10 nM by calculating the concentration based off the fragment size (base pairs) and the concentration (ng/μl) of the libraries. Sequencing was performed on an Illumina NovaSeq 6000 (UPMC Genome Center). Libraries were sequenced on an S2 flow cell with a target of ~50 million reads per sample.

### Analysis of bulk RNA‐seq data

PBMC RNA gene expression was analyzed by RNA sequencing (RNA‐seq). We then used three approaches to examine the RNA‐seq data: Examining genes that were differentially expressed between stable and progressive patients using DESeq2, examining pathways regulated by these genes with uncorrected *P* < 0.05 using Gene Ontology (GO). False discovery rate (FDR) <5% was considered statistically significant. Genes were then clustered by meeting an expression threshold for detection using Cluster 3.0 and Java TreeView. The uncorrected *P* value was used to identify potential candidate genes.

### Single‐cell RNA sequencing of PBMC

PBMC were collected from patients and HCs and stored frozen in 10% dimethyl sulfoxide. Frozen PBMC were thawed in batches of 10 samples, including two from HCs and three or four each of samples from patients with progressive SSc‐ILD and stable SSc‐ILD. Samples were labeled with barcoded antibodies for multiplexing samples (10X Genomics),^14^ as described previously.^15^ Targeting 5,000 cells sample, cells were partitioned, and single‐cell libraries prepared using the V3 3′ chemistry (10X Genomics), according to the 10X Genomics protocol. Libraries were sequenced, and sequences demultiplexed and aligned in Cell Ranger (10X Genomics).

### Analysis of single‐cell RNA‐seq data

Cell gene expression matrixes were generated from the aligned sequence data. The Model‐based Analysis of Single‐cell Transcriptomics (MAST) statistical algorithm feature with adjusted *P* < 0.05 in Seurat version 4 was used to discover differentially expressed genes in the single‐cell RNA‐seq (scRNA‐seq) data comparing cells from patients with progressive SSc‐ILD with cells from patients with stable SSc‐ILD cluster by cluster. Cells were plotted by transcriptome using the uniform manifold approximation and projection (UMAP) algorithm for dimensionality reduction, annotated using Azimuth (Figure 3A),^15^ and confirmed cluster identities using known marker genes (Supplementary Figure 8). We used Kruskal‐Wallis test to compare samples from control, stable SSc‐ILD, and progressive SSc‐ILD. Dunn's test was performed following Kruskal‐Wallis to identify which specific groups differ.

## RESULTS

### Male sex and shorter disease duration associated with progressive SSc‐ILD

Ages were well‐balanced between patients with progressive SSc‐ILD (56.59 years) and patients with stable SSc‐ILD (58.79 years). Progressive SSc‐ILD was strongly skewed toward patients who were men and with shorter disease duration (Table 1). Men with progressive disease represented 46.5% of patients with progressive SSc‐ILD but only 15.4% of patients with stable SSc‐ILD (*P* = 0.0038). Disease duration was significantly shorter in patients with progressive disease (8.86 years) compared with patients with stable disease (15.55 years; *P* = 0.01242). As would be expected from the criteria for progressive disease, the baseline FVC was significantly lower in patients with progressive SSc‐ILD (74.1% predicted) compared with patients with stable SSc‐ILD (60.7% predicted).

**Table 1:** Patient demographics, clinical features, serum biomarkers, and autoantibodies*

	Stable (n = 39)	Progressive (n = 43)	Nominal *P*‐value^a^
Sex, male	6	20	**0.0038**
Age, y	58.79	56.59	0.4777
Disease duration,^b^ y	15.55	8.86	**0.01242**
Limited/diffuse^c^	26/9	22/12	0.4251
Baseline predicted FVC, %	70.1	60.7	**<0.0001**
MRSS^d^	4.62	6.03	0.08726
CRP	5053.7	9663.3	**0.0394**
KL‐6	36.0	119.1	**0.00044**
SP‐D	11.0	13.8	0.4965
CPK	66.2	70.8	0.88866
Autoantibodies^e^			
anti‐Scl‐70	6	14	0.0783
anti‐Th/To	7	3	0.1796
anti‐RNAPol3	5	4	0.7292
anti‐centromere	7	3	0.1796
anti‐Ku	1	1	1.000
anti‐PM/Scl	3	4	1.000
anti‐U3	2	1	0.6018
anti‐U1/U2 RNP	4	1	0.1842
anti‐Ro	1	4	0.3622

The bolded values are the *p*‐values that reached statistical significance (*p *< 0.05).

* One patient showed autoantibodies to both Ku and RNApol3. Bold *P* values indicate statistical significance. CPK, creatine phosphokinase; CRP, C‐reactive protein; FVC, forced vital capacity; KL‐6, Krebs von den Lungen 6; MRSS, modified Rodnan Skin Score; RNP, ribonucleoprotein; SP‐D, surfactant protein‐D.

^a^ Two‐sided Mann‐Whitney, or two‐sided Fisher's exact test for binomial values.

^b^ Patients in stable disease group, n = 31; patients in progressive disease group, n = 32.

^c^ Patients in stable disease group, n = 35; patients in progressive disease group, n = 34.

^d^ Patients in stable disease group, n = 32; patients in progressive disease group, n = 32.

^e^ For all autoantibodies measures, patients in stable disease group (n = 38), patients in progressive disease group (n = 42).

### Elevated CRP and KL‐6 levels in patients with progressive SSc‐ILD

We examined the sera to see if KL‐6, CRP, SP‐D, or CPK levels were predictive for progressive SSc‐ILD. Both KL‐6 and CRP levels were statistically significantly higher in patients with progressive SSc‐ILD compared with patients with stable SSc‐ILD (Table 1, Figure 1B). Notably, there was little correlation between CRP and KL‐6 levels (*r* ^*2*^ = 0.0178; *P* > 0.05), suggesting that these two biomarkers provide somewhat complementary information regarding prognosis. SP‐D levels failed to show a difference between patients with progressive SSc‐ILD and patients with stable SSc‐ILD; however, many of the SP‐D measures were at the limit of detection, perhaps explaining why these results are not consistent with some past studies. CPK, found in an early analysis of the GENOSIS cohort to be protective for the presence of SSc‐ILD^9^ and was later shown to be weakly associated with a lower baseline FVC after adjustment for follow up time,^10^ did not show a significant difference between patients with progressive SSc‐ILD and patients with stable SSc‐ILD in our cohort.

In addition, we looked at autoantibodies in the sera of these patients, detectable in 65 of 80 patients. Patients with anti‐Scl‐70 autoantibodies had a strong trend toward having progressive disease (*P* = 0.0783), whereas patients with anti‐centromere (ACA), anti‐U1 or anti‐U2, and anti‐Th or anti‐To antibodies were associated with less progressive disease (*P* = 0.1796 for each; Table 1). Other autoantibody specificities did not associate with patients with stable SSc‐ILD or patients with progressive SSc‐ILD. These autoantibody and serum data indicated that our designation of patients with stable SSc‐ILD and patients with progressive SSc‐ILD were consistent with previous studies of patients with SSc‐ILD.

### Monocyte‐like cells increased expression of lipoxygenase homology domains 1

We examined the most differentially expressed genes (DEGs) between patients with progressive SSc‐ILD and patients with stable SSc‐ILD and ranked these by uncorrected *P* < 0.05 (Supplementary Table 1). Although none of these genes reached a corrected *P* value or FDR of less than five percent, several genes appeared potentially of interest as biomarkers based on their increased expression in SSc‐ILD monocyte‐like myeloid cells in our previously reported single‐cell RNA sequencing (scRNA‐seq) data. Among the genes elevated in patients with progressive SSc‐ILD, *S100A12* stood out for its previously reported role in patients with progressive IPF,^11^ highlighting its potential as a shared biomarker of fibrotic lung disease. Similarly, *S100A9* and *FPR2* were up‐regulated, associated with immune activation, which may suggest a proinflammatory state contributing to disease progression (Supplementary Figure 1). Notably, *LOXHD1*, which also showed upregulation in our scRNA‐seq data set (Figure 1C, Supplementary Figure 1), may also reflect pro‐inflammatory programming relevant to fibrotic progression. Among genes with nominal significance (uncorrected *P* < 0.05), *MMP9* emerged as one of the most up‐regulated in patients with progressive SSc‐ILD versus patients with stable SSc‐ILD, showing an 8.76‐fold increase (Supplementary Figure 2, Supplementary Table 1). Matrix metalloproteinase‐9 (MMP9) is a known biomarker for SSc‐ILD and may reflect ongoing tissue remodeling and fibrotic activity.^12^ We have previously described markedly up‐regulated MMP9 by SPP1 macrophages,^3^, ^4^ a macrophage subset associated with increased SPP1 expression and fibrosis, in lungs from patients with SSc‐ILD (Supplementary Figure 2). SPP1 also appeared elevated in the PBMCs from patients with progressive SSc‐ILD, although its expression level was lower than the threshold that we employed to filter noise in gene expression. *SPP1* is a gene known to be up‐regulated by profibrotic macrophages in patients with IPF and patients with SSc‐ILD.^3^, ^4^ Ras homolog gene family, member B (RHOB) was also seen up‐regulated.

We evaluated whether genes with nominal significance were enriched in specific biologic pathways. GO indicated several statistically significant pathways (Supplementary Table 2). Several enriched pathways were inflammation‐related, including inflammatory response (6.13‐fold), chronic inflammatory response (65.37‐fold), respiratory burst (44‐fold), response to lipopolysaccharide (LPS) (7.06‐fold), and neutrophil chemotaxis (17.16‐fold). Key genes involved included *CCL4L2*, *F12*, *FPR2*, *S100A8*, *FOS*, *LYN*, *S100A9*, *CXCL1*, *VNN1*, and *S100A12*.

We also examined down‐regulated genes (Supplementary Table 3). Again, none of these genes reached an FDR of less than five percent. However, several T cell receptor genes and the *IL10RA* gene showed decreased expression in T cells and monocyte‐like macrophages, respectively, in lungs from patients with SSc‐ILD (Supplementary Figure 3). Pathway analysis of down‐regulated genes indicated pathways centered around nucleic acid and messenger RNA metabolism (Supplementary Table 3).

### Inflammatory gene cluster more highly expressed by PBMCs from patients with progressive disease

**Figure 2:**
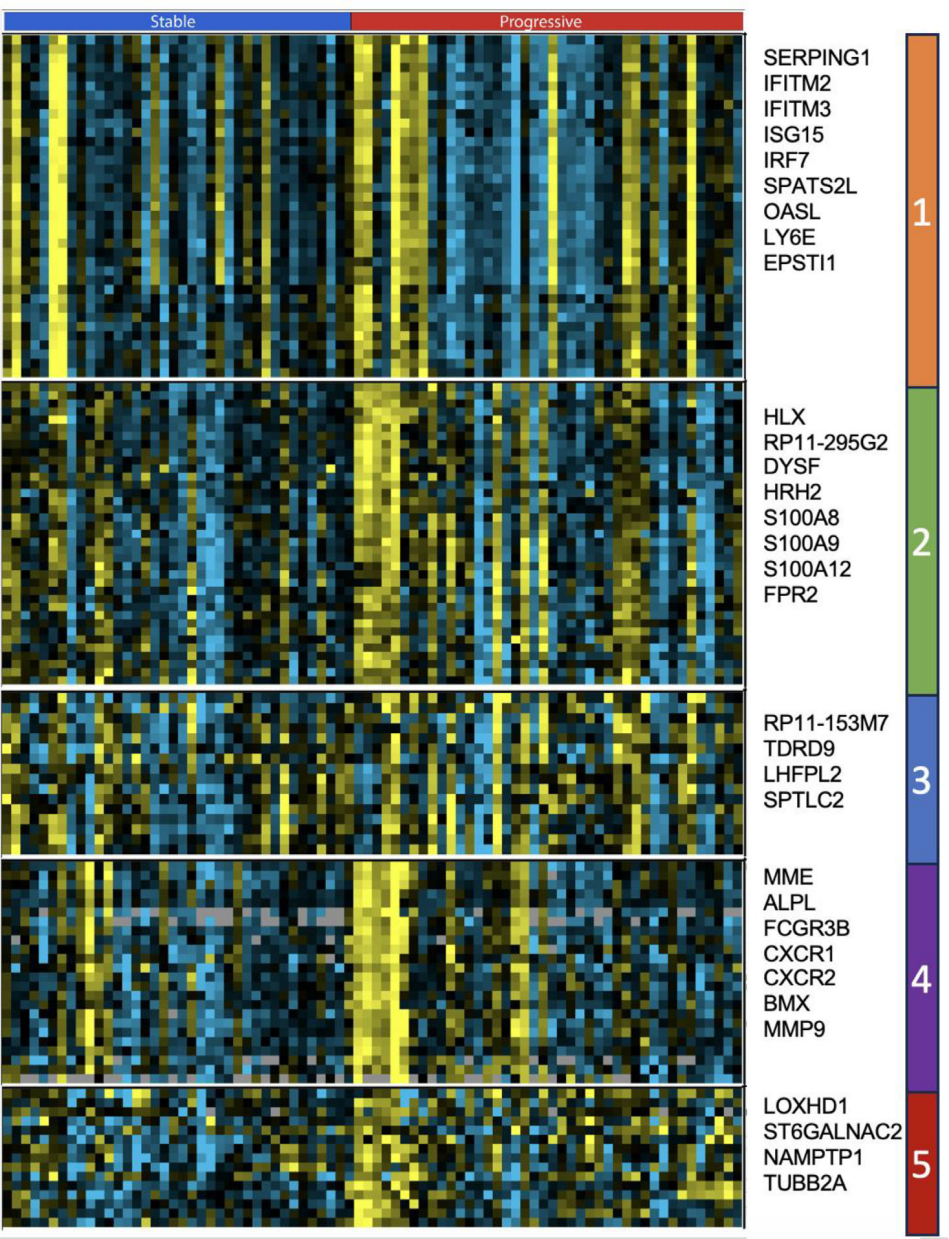
Gene cluster heatmap showing different clusters according to their inflammatory gene signature from PBMCs. PBMC, peripheral blood mononuclear cells. Color figure can be viewed in the online issue, which is available at http://onlinelibrary.wiley.com/doi/10.1002/acr.25619/abstract.

Clustering of genes revealed several groups of highly correlated genes. Most prominently, IFN‐regulated genes were expressed by a subset of patients, but none of these genes were up‐regulated in patients with progressive SSc‐ILD (Figure 2, cluster 1). A second cluster contained *S100A8*, *S100A9*, *S100A12*, and *FPR2*, genes up‐regulated in patients with progressive disease and highlighted in the GO pathway analysis above (Figure 2, cluster 2). The up‐regulated gene most highly correlated with patients with progressive disease was *RP11‐153M7* (a *TLR2*‐related pseudogene), clustered with other genes, but none of the other genes in this cluster distinguished progressive disease (Figure 2, cluster 3). A fourth cluster included *MMP9*, *BMX*, *CXCR1*, *CXCR2*, and *FCGR3B/CD16B* genes. These genes were both more highly expressed or trended strongly toward higher expression in PBMCs from patients with progressive SSc‐ILD. A fifth cluster included *LOXHD1* and *TUBB2A*, both more highly expressed in PBMCs from patients with progressive SSc‐ILD (Supplementary Figure 4 for full gene list).

Several additional clusters of genes showed higher expression in patients with progressive SSc‐ILD. A cluster of Y chromosome genes were easily recognized as only expressed by male patients, and a cluster of hemoglobin and other genes associated with red blood cells appeared to be coming from reticulocytes (Supplementary Figure 5), possibly related to hypoxemia or increased bleeding in these patients often known to have gastric antral vascular telangiectasia. In addition, a cluster included the chitinase genes *CHIT1* and *CHI3L1* that encode serum biomarkers of fibrotic lung disease.^13^, ^14^ These genes trended toward higher expression in patients with SSc‐ILD, *CHI3L1* meeting an uncorrected *P* < 0.05 (Supplementary Figures 6A and 7). Finally, a cluster included genes significantly (*RHOB*, *SAT1*, and *C5AR1*) (Supplementary Figure 6B) or strongly trending higher (*FTH1*, *PLAUR*, and *SERTD3*) in PBMCs from patients with progressive SSc‐ILD (Supplementary Figure 6A). Chitinase 1 (CHIT1) and chitinase‐3‐like protein 1 (CHI3L1) were both much more highly expressed in SPP1 macrophages in patients with SSc‐ILD compared with HCs in scRNA‐seq data from our previous scRNA‐seq datasets from lungs from patients with SSc.^3^ A large collection of genes was down‐regulated in patients with stable SSc‐ILD, including *CRYBB2P1*, the most statistically significantly down‐regulated gene (Supplementary Table 4).

### Marker genes of progressive SSc‐ILD by single‐cell RNA‐seq of PBMCs

**Figure 3:**
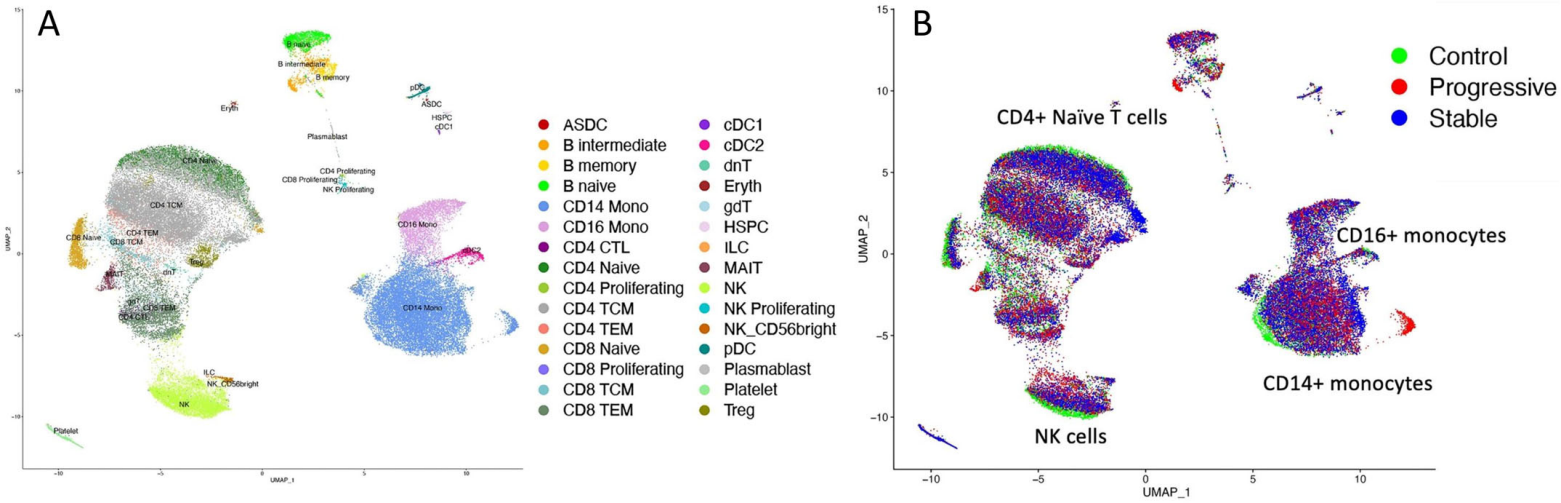
(A) UMAP of PBMC for progressive and stable SSc‐ILD patients and controls colored by cell‐type. (B) UMAP of peripheral blood NK cells, T cells, and monocytes colored by patient population (eg, progressive disease, stable disease, and control). Shifts within clusters between patients with progressive SSc‐ILD and patients with stable SSc‐ILD and control participants were observed. ASDC, AXL+Siglec‐6+ dendritic cells; cDC, classic dendritic cells; dnT, double negtive T cells; Eryth, erythrocytes; gdT, gamma‐delta T cells; HSPC, hemopoietic stem and progenitor cells; ILC, innate lymphoid cells; MAIT, mucosal‐associated invariant T; NK, natural killer; PBMC, peripheral blood mononuclear cells; pDC, plasmacytoid dendritic cells; SSc‐ILD, scleroderma with interstitial lung disease; TCM, T central memory; TEM, T effector memory cells.

**Figure 4:**
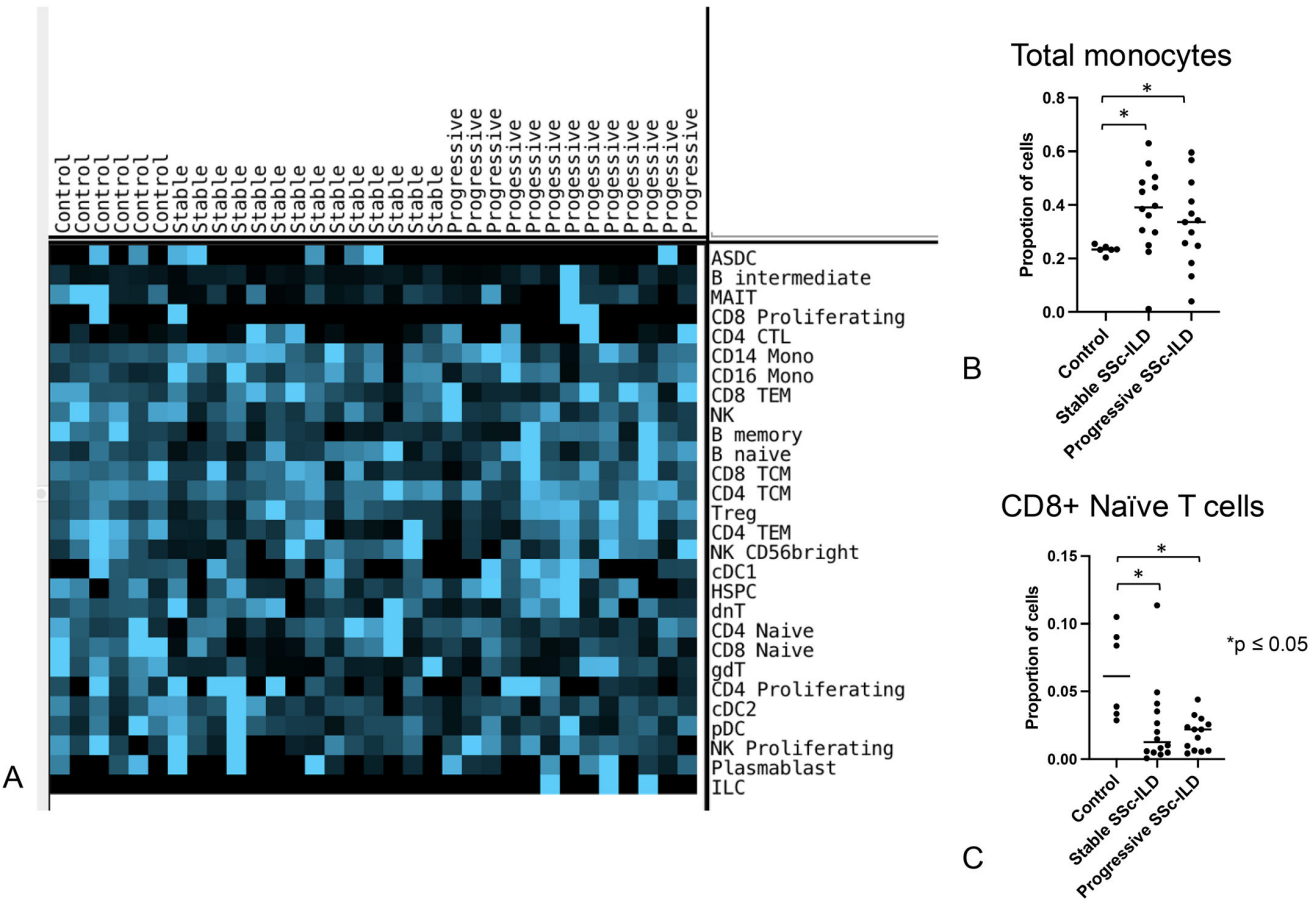
(A) Pseudobulk expression heatmap of cell types according to patient group. (B) Proportion of total monocytes in control, stable SSc‐ILD, and progressive SSc‐ILD groups each patient population. A significant overall difference was detected (*P* = 0.043) using Kruskal‐Wallis test; the asterisk indicates significant difference between control participants and patients with SSc‐ILD (stable disease and progressive disease combined) (C) Proportion of CD8+ naive T cells in each patient population. A significant overall difference was detected (*P* = 0.015); post hoc test showed significant differences between control participants and patients with stable SSc‐ILD and control participants and patients with progressive SSc‐ILD, but not between patients with stable SSc‐ILD and patients with progressive SSc‐ILD. *P ≤ 0.05. ASDC, AXL+ Siglec‐6+ dendritic cells; cDC, classic dendritic cells; dnT, double negative T cells; gdT, gamma‐delta T cells; HSPC, hemopoietic stem and progenitor cells; ILC, innate lymphoid cells; MAIT, mucosal‐associated invariant T; NK, natural killer; pDC, plasmacytoid dendritic cells; SSc‐ILD, scleroderma with interstitial lung disease; TCM, T central memory cells; TEM, T effector memory cells. Color figure can be viewed in the online issue, which is available at http://onlinelibrary.wiley.com/doi/10.1002/acr.25619/abstract.

We analyzed PBMCs from HCs (n = 6), patients with stable SSc‐ILD (n = 14), or patients with progressive SSc‐ILD (n = 13) by scRNA‐seq (Figure 3A) and confirmed cluster identities using known marker genes (Supplementary Figure 8). We first looked at the proportions of cells in the different patient populations. Clustering by cell proportion did not reveal any striking associations between the proportions of cells in the different patient populations (Figure 4A). Of the cell populations, CD8+ naive T cells and monocytes (combining CD14+ and CD16+) showed significant differences in cell proportions across the subsets of HCs, patients with stable SSc‐ILD, and patients with progressive SSc‐ILD (Figure 4B and C). However, these differences were driven by comparisons between the subsets of HCs and patients with SSc‐ILD, with significant changes observed between patients with stable SSc‐ILD versus HC and patients with progressive SSc‐ILD versus HC for CD8+ naive T cells. No significant differences were detected between patients with stable SSc‐ILD and patients with progressive SSc‐ILD.

Next, we examined gene expression on Uniform Manifold Approximation and Projection (UMAP) plots by sample (not shown) and by disease status (Figure 3B). The latter showed shifts in the cell populations within clusters. Comparing PBMC transcriptomes from HCs with patients with SSc‐ILD, two clusters of cells from patients with progressive SSc‐ILD stood out separately. However, these clusters were only seen in one patient (not shown) and so were not representative of consistent changes in gene expression associated with patients with progressive SSc‐ILD. Closer examination also suggested that there were shifts within clusters between samples from patients with progressive SSc‐ILD and patients with stable SSc‐ILD, most notably in CD14+ monocytes, CD4+ naive T cells, and NK cells (Figure 4).

### DEGs in CD14+ monocytes from patients with progressive SSc‐ILD

We systematically examined changes in gene expression associated with cells (scRNA‐seq) from patients with progressive SSc‐ILD compared with patients with stable SSc‐ILD.^15^ We then examined data by sample (pseudobulk) to identify biomarkers associated with PBMC cell populations from individual HCs, patients with stable SSc‐ILD, and patients with progressive SSc‐ILD that might be useful as robust biomarkers of disease progression. CD14+ monocytes showed a series of genes that met criteria for differential expression as well as a difference comparing patients with stable SSc‐ILD with patients with progressive SSc‐ILD (Supplementary Table 5).

**Figure 5:**
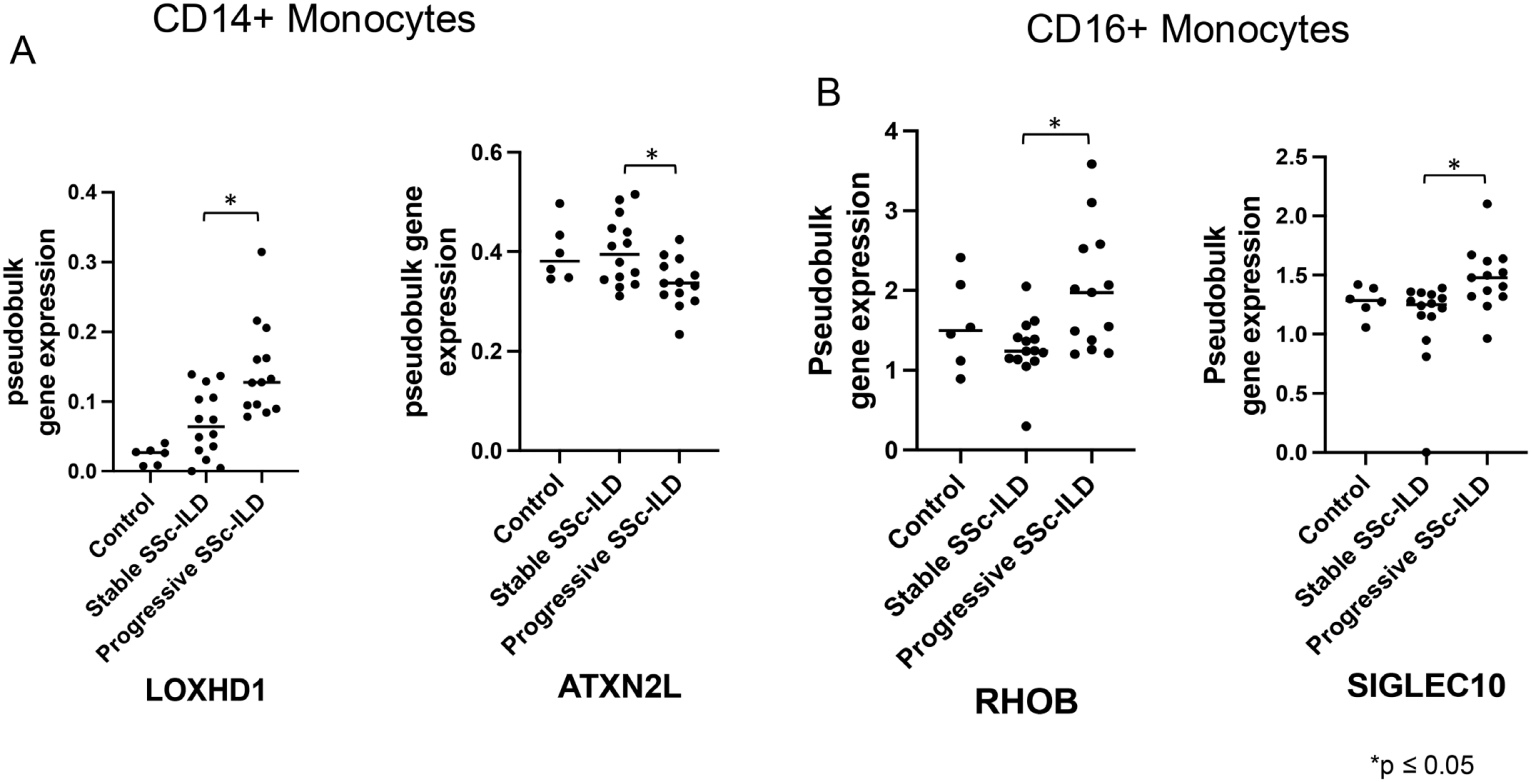
(A) CD14+ monocyte genes (*LOXHD1*, *ATXN2L*) showing significant differences (using MAST and Kruskal‐Wallis statistics) in control participants, patients with stable SSc‐ILD, and patients with progressive SSc‐ILD. (B) CD16+ monocyte genes (*RHOB*, *SIGLEC10*) showing significant differences based on comparing cells (MAST *P*_*adj*_ < 0.05) and samples (*P* < 0.05, Kruskal‐Wallis) in control participants, patients with stable SSc‐ILD, and patients with progressive SSc‐ILD. *P ≤ 0.05. SSc‐ILD, scleroderma with interstitial lung disease.

Several also showed statistically significant differences among HC, patients with stable disease, and patients with progressive disease: *LOXHD1*, *ATXN2L*, *EIF4E*, *PIK3CG*, *PRELID1* (Figure 5A, Supplementary Figure 9) and specific differences between patients with stable disease and patients with progressive disease (Dunn's test). *LOXHD1* was the only gene among these to show differential expression between patients with stable SSc‐ILD and patients with progressive SSc‐ILD in the bulk RNA‐seq data, suggesting a potential role in patients with monocyte‐driven progressive SSc‐ILD. Although *ATXN2L* did not show significant differences within individual groups, it demonstrated a statistically significant decrease in expression in the bulk RNA‐seq dataset between patients with progressive SSc‐ILD compared with patients with stable SSc‐ILD, suggesting a potential role in disease progression. *ATXN2L* is also down‐regulated in all macrophage populations in the lungs of patients with SSc‐ILD (Supplementary Figure 10).

Examining genes associated with patients with progressive SSc‐ILD in PBMC in bulk RNA‐seq, *S100A8*, *S100A9*, and *S100A12*, all highly expressed but not highly up‐regulated in the SPP1 macrophages in lungs of patients with SSc‐ILD, were not up‐regulated in the PBMC CD14+ monocytes of patients with progressive SSc‐ILD (not shown). The reason for this difference is uncertain. *CHIT1*, *CHI3L1*, and *MMP9*, although strongly up‐regulated in SPP1 macrophages in lungs of patients with SSc‐ILD, were not reliably detected in CD14+ monocytes in the scRNA‐seq PBMC data (not shown). Thus, *LOXHD1* and *ATXN2L* appeared to be the most consistent biomarkers of progressive SSc‐ILD because they were up‐regulated (*LOXHD1*) or down‐regulated (*ATXN2L*) in CD14+ cells on scRNA‐seq and validated in the bulk RNA‐seq dataset.

We also examined RNA gene changes in patients with SSc‐ILD, including both samples from patients with progressive disease and patients with stable disease compared with control PBMCs. We highlight several genes of particular interest. Both *NFKB1* and *RELB*, parts of the classical and alternative NF‐κB signaling pathways,^16^ were respectively down‐regulated in PBMCs from patients with SSc‐ILD (Supplementary Figure 11). *NFKB1* is down‐regulated in multiple cell populations in the lungs of patients with SSc‐ILD (Supplementary Figure 12). *OSM*, *CASS4*, *PRDM1*, and *RAPGEF1* were also down‐regulated in lung CD14+ monocytes from patients with SSc‐ILD compared with HCs (Supplementary Figure 13).

### DEGs in CD16+ monocytes from patients with progressive SSc‐ILD

Using a similar approach, we identified a set of genes in CD16+ monocytes associated with patients with progressive SSc‐ILD versus patients with stable SSc‐ILD in scRNA‐seq. Four genes, *RHOB*, *SIGLEC10*, *RHOC*, and *GRK3*, showed consistent differential expression between cells from patients with progressive SSc‐ILD and patients with stable SSc‐ILD (Figure 5B and Supplementary Figure 14); supported by multiple statistical approaches comparing samples from patients with progressive SSc‐ILD with patients with stable SSc‐ILD (Figure 5B). Of these genes, only *RHOB* was increased in patients with progressive SSc‐ILD in the PBMC bulk RNA‐seq data (Figure 5B, Supplementary Figure 15). None of these genes were up‐regulated in CD14+ monocytes in the lungs of patients with SSc‐ILD compared with control participants. Reexamining the PBMC CD14+ monocyte data, *RHOB* was also increased in CD14+ monocytes from patients with progressive SSc‐ILD (*t*‐test; *P* = 0.028), although failing to meet statistical significance in the Kruskal‐Wallis test. These findings suggest RHOB may serve as a robust peripheral biomarker of disease progression.

Regarding markers identified in CD14+ monocytes, *LOXHD1* showed a trend toward increased expression in CD16+ monocytes but its level of expression was much lower than in CD14+ monocytes. *ATXN2L* failed to show a significant down‐regulation in CD16+ monocytes from patents with progressive SSc‐ILD. Thus, *RHOB* was the most convincing biomarker of patients with progressive SSc‐ILD because it alone was up‐regulated in CD16+ and CD14+ cells on scRNA‐seq and was validated in the bulk RNA‐seq dataset.

### DEGs in NK and CD4+ naive T cells from patients with progressive SSc‐ILD

NK and CD4+ naive T cells exhibited separation of cells (scRNA‐seq) between patients with progressive SSc‐ILD and patients with stable SSc‐ILD (Figure 3). Eight NK cell genes were significantly differentially expressed between progressive and stable samples (Supplementary Table 5). Among these, three genes—*NCR3*, *RHD*, and *MT‐CO2*— also showed significant differences across control participants, patients with progressive SSc‐ILD, and patients with stable SSc‐ILD, specifically between samples from patients with progressive SSc‐ILD and patients with stable SSc‐ILD by Dunn's test (Supplementary Figure 15; Supplementary Table 5). However, none of these were detectably different in the bulk RNA‐seq data (Supplementary Figure 16). In prior scRNA‐seq of lungs from patients with SSc‐ILD, *RHD* was expressed at very low levels but enriched in NK cells; *NCR3* was most highly expressed in lungs from HCs compared with patients with SSc‐ILD; and *MT‐CO2* was broadly expressed but decreased in lungs from patients with SSc‐ILD relative to HCs (Supplementary Figure 16). NK cell effector genes previously reported to be up‐regulated in lung NK cells from patients with SSc‐ILD—*GZMB*, *IFNG* and *AREG* ^6^—were not increased in scRNA‐seq from PBMC NK cells or in patients with progressive disease in this dataset (not shown).

Ten naive CD4+ T cell genes were statistically significant when comparing cells from patients with progressive SSc‐ILD versus patients with stable SSc‐ILD (Supplementary Table 5). Two NK cell genes, *CHI3L2* and *MT‐CO2*, also showed significant differences when comparing HCs, patients with progressive SSc‐ILD, and patients with stable SSc‐ILD; and also between patients with progressive SSc‐ILD and patients with stable SSc‐ILD by Dunn's test (Supplementary Figure 16). Both genes showed a weak trend toward differences between patients with progressive SSc‐ILD and patients with stable SSc‐ILD in bulk RNA‐seq. As noted above, *MT‐CO2* was also significantly decreased in NK cells from PBMCs as well as lungs from multiple populations with SSc‐ILD. *CHI3L2* did not show up‐regulated expression in lung T cells and was more highly expressed and down‐regulated in lung alveolar type 2 cells and myofibroblasts from patients with SSc‐ILD (Supplementary Figure 17).

## DISCUSSION

Monocyte expression of LOXHD1 stands out as the one gene most highly predictive of progressive SSc‐ILD. Most studies of LOXHD1 have focused on its role in hearing loss. It is part of the stereociliary complex protruding from cochlear hair cells and is required for mechanotransduction through membrane structures referred to as tip links. Mutation of LOXHD1 affects mechanotransduction but not the structure of tip links.^17^ However, a more recent study suggests another function. Increased expression of an alternative, shorter transcript has been associated with disordered cytoskeleton and decreased metastatic potential.^18^

Our findings are consistent with past studies, indicating that male sex, KL‐6, and CRP levels are predictors of progressive disease. KL‐6 was seen in one study of a mixed population with SSc‐ILD and Mixed Connective Tissue Disease‐associated interstitial lung disease (MCTD‐ILD) as predictive of progressive disease^19^ and in a combined analysis of patients with SSc‐ILD.^20^ Male sex has previously been identified as a risk factor for progressive SSc‐ILD^20^ as has CRP levels.^21^, ^22^

In our study cohort, anti‐Scl‐70 was seen more often in progressive disease, and ACA more commonly in stable SSc‐ILD. Anti‐Scl‐70 has been associated with the presence of SSc‐ILD in multiple studies^23^, ^24^, ^25^ and also with progressive SSc‐ILD,^10^ whereas ACAs were negatively associated with SSc‐ILD and trended toward protection from progressive disease.^10^, ^23^, ^24^, ^25^ Another recent study found no relationship between anti‐Scl‐70 and progressive decline in FVC but did see a decline in patients with anti‐Ro antibodies.^26^ Variability in studies examining the relationship between anti‐Scl‐70 and SSc‐ILD have been related to the method for its detection.^27^ We saw anti‐Ro in more patients with progressive SSc‐ILD than patients with stable SSc‐ILD but was observed too infrequently to be informative about its potential association with progressive disease. Our sample size may have been too small to show statistically significant associations between progressive ILD and these autoantibodies.

Down‐regulated expression of ATXN2L and up‐regulated expression of RHOB were also markers in patients with progressive SSc‐ILD in our scRNA‐seq dataset. We validated dysregulated expressions of *ATXN2L* and *RHOB* in the bulk RNA‐seq data. Rho proteins are important in regulating cell cytoskeleton through actin fibers, which are also the structural proteins of stereocilia^28^ and potentially linking the function of RHOB and LOXHD1. Although RHOB is not required for macrophage chemotaxis,^29^ it is tempting to speculate that the role of both LOXHD1 and RHOB might be related to macrophage locomotion. ATXNL2 along with ATXN2 regulate protein translation in circadian rhythm,^30^ but ATXNL2 is also part of a macrophage signature that includes SPP1 predicting clinical features in patients with meningioma^31^ and is widely expressed by cells in the lungs, suggesting other roles.

S100A12, CHI3L1, and MMP9 are part of clusters or pathways associated with progressive SSc‐ILD in bulk RNA‐seq but were not up‐regulated (S100A12) or not reliably detected (MMP9/CHI3L1) in monocytes in the scRNA‐seq dataset. Past serum data strongly support these as PBMC bulk RNA‐seq biomarkers. Serum S100A12 is a prognostic biomarker in IPF.^32^ Serum levels of CHI3L1 (previously known as YKL‐40) are higher in patients with SSc‐ILD^33^ and patients with IPF.^34^ CHI3L1 activates profibrotic macrophages through CRTH2, suggesting a direct role in pathogenesis.^35^ MMP9 is elevated in patients with IPF^36^ and patients with SSc,^37^ but has not been studied specifically in patients with SSc‐ILD. Thus, the gene clusters represented by these genes in bulk RNA‐seq likely represent true markers of progressive disease.

The origin of SPP1 macrophages in SSc‐ILD lungs remains uncertain, ie circulating monocytes or resident lung macrophage populations. Significantly, several genes that show profoundly up‐regulated expression by SPP1 macrophages in SSc‐ILD lungs (SPP1, CHIT1, CHI3L1 and MMP9);^2^, ^3^ are expressed at very low levels by SSc‐ILD PBMC monocytes. Studies of patients undergoing bronchoalveolar lavage or retransplant after a transplant suggest that most alveolar macrophages are donor‐derived.^38^, ^39^ However, other studies have shown that recipient macrophages largely replace alveolar macrophages after transplant, suggesting that most alveolar macrophages are derived from monocytes.^40^ Our previous scRNA‐seq data from lungs from patients with SSc‐ILD and patients with IPF show that alveolar and profibrotic SPP1‐macrophages, but not monocyte‐like macrophages are actively proliferating.^2^, ^3^ Collectively these observations suggest that monocytes in the periphery do not differentiate in the circulation to profibrotic SPP1 macrophages (and then migrate into the lungs), but that circulating monocytes infiltrate into lungs as monocyte‐like macrophages, potentially differentiating into SPP1 macrophages. However, our observations here suggest that either monocytes undergo profound changes in gene expression after migrating into SSc‐ILD lung tissues or that the SPP1 macrophages in SSc‐ILD derive from proliferation of resident macrophage populations.

CD14+ monocyte counts have been shown as prognostic for transplant‐free survival in IPF but not non‐IPF fibrotic lung disease (including SSc‐ILD).^41^, ^42^ Partially consistent with these observations, we detected increased monocyte counts in SSc‐ILD compared to control samples, though not showing even a trend toward increased monocytes in patients with progressive SSc‐ILD compared with patients with stable SSc‐ILD. Although our data suggest that alterations in monocyte counts are associated with the development of SSc‐ILD because our control groups did not include a group of SSc patients without ILD, altered monocyte counts could be related to the development of SSc rather than to the development of SSc‐ILD. A recent study of monocytes in IPF and hypersensitivity pneumonitis appears to show some common features with altered gene expression in SSc‐ILD monocytes, including increased expression of S100A8 and S100A9.^43^

Recently, a group stratified monocyte populations from patients with SSc into three groups based on gene expression.^44^ One of these groups (group C) associated with more severe lung disease appears to be similar to the IFN‐regulated gene expression we reported previously^7^ and appears again in this dataset. A previous study showed that the presence of the IFN signature in patients with SSc PBMC correlates with plasma levels of gene products of two IFN‐regulated genes CXCL10 and CXCL11.^45^ ITAC/CXCL11 but not IP‐10/CXCL10 correlated weakly with FVC. Our dataset here failed to show a significant correlation between progressive SSc‐ILD and the IFN signature.

A recent study of proteomic biomarkers of progressive SSc‐ILD identified 29 differentially expressed proteins, although not statistically significantly different after adjustment for multiple comparisons. One of these, ZNF500, showed an unadjusted *P* < 0.05 and direction, that is, decreased expression in progressive patients, in our bulk RNA‐seq data (unadjusted *P* = 0.0148).

Compared with other longitudinal studies of patients with SSc‐ILD, the current study defined patients with progressive disease over a relatively short follow up period. This design identifies patients with progressive disease at the time of blood sampling rather than long‐term prognosis. This was felt to be important for making decisions regarding treatment but also felt to be more likely to detect markers of disease activity. In addition, long‐term prognosis is increasingly affected by treatments because medications affecting the course of the disease have become available. Notably, the average disease duration was shorter in our patient group with progressive SSc‐ILD than in our patient group with stable SSc‐ILD despite having lower average baseline FVC. These two observations may be related because previous trajectory analyses have suggested that a subgroup of patients with SSc with low FVC show a rapid decline in FVC.^46^ Because our patients were not matched for disease duration or baseline FVC, we cannot exclude the possibility that these variables are affecting the biomarkers we have described. Because this is a relatively small study, it should be viewed as exploratory. For this reason, some comparison between progressive versus regressive SSc‐ILD were assessed by uncorrected *P* values. However, these uncorrected *P* values led to significant correlations with pathways, suggesting they detected underlying mechanisms of monocyte activation. Despite study limitations with validation these biomarkers hold promise to assist with the clinically challenging assessment of SSc‐ILD.

## AUTHOR CONTRIBUTIONS

All authors contributed to at least one of the following manuscript preparation roles: conceptualization AND/OR methodology, software, investigation, formal analysis, data curation, visualization, and validation AND drafting or reviewing/editing the final draft. As corresponding author, Dr Lafyatis confirms that all authors have provided the final approval of the version to be published and takes responsibility for the affirmations regarding article submission (eg, not under consideration by another journal), the integrity of the data presented, and the statements regarding compliance with institutional review board/Declaration of Helsinki requirements.

## ROLE OF THE STUDY SPONSOR

Mitsubishi Inc had no role in the study design or in the collection, analysis, or interpretation of the data, the writing of the manuscript, or the decision to submit the manuscript for publication. Publication of this article was not contingent upon approval by Mitsubishi Inc.

## Supporting information


**Disclosure Form**:

**Data S1** Supporting Information

**Figure S1.** Differential expressed genes of monocytes by single cell RNA‐seq of S100A12, S100A9, FPR2, and LOXHD1 between SSc‐ILD and control (HC/NOR).
**Figure S2.** Upregulation of MMP9 in SSc‐ILD SPP1 macrophages by single cell RNA‐seq. Increased MMP9 gene expression in progressive disease compared to stable (p‐value=0.049. Horizontal line in scatterplot denotes the average.
**Figure S3.** T cell receptors (TRAV21, TRBV19) and IL10RA showed decreased expression in scRNA‐seq from scleroderma cohort.
**Figure S4.** Full gene list for each cluster group.
**Figure S5.** A cluster of Y chromosome genes and a cluster of hemoglobin and other genes associated with red blood cells from reticulocytes. They may be related to vascular changes observed in SSc (e.g. telangiectasia, gastric antral vascular ectasia).
**Figure S6.** A) CHIT1 and CHI3L1 and their associated genes. Chitinase genes encode serum biomarkers of fibrotic lung disease and trended toward higher expression in SSc‐ILD. B) RHOB and SAT1 and their associated genes.
**Figure S7.** A) Pseudobulk RNA expression of CHI3L1 between progressive and stable SSc‐ILD. B) Increased single cell RNA expression of CHIT1 and CHI3L1 in SSc‐ILD SPP1 macrophages compared to control (NOR). P‐value is uncorrected and horizontal line in scatterplot denotes the average.
**Figure S8.** Feature Plots showing known gene markers to confirm identities of different clusters in the UMAP (Figure 3)
**Figure S9.** EIF4E, PIK3CG, and PRELID1 gene expression in CD14+ monocytes between control, stable, and progressive SSc‐ILD. Horizontal line in scatterplot denotes the average.
**Figure S10.** ATXN2L gene expression in different lung cell types between control and SSc.
**Figure S11.** RELB and NFKB1 showing decreased expression in SSc‐ILD CD14+ monocytes (by sample FDR<0.05)
**Figure S12.** NFKB1 is decreased in multiple cell populations in SSc‐ILD lungs compared to controls.
**Figure S13.** OSM, CASS4, PRDM1, RAPGEF1 show decreased expression in SSc‐ILD CD14+ monocytes (by sample FDR<0.05)
**Figure S14.** RHOC and GRK3 pseudobulk gene expression show significant differences between progressive versus stable disease. Horizontal line in scatterplot denotes the average.
**Figure S15.** Expression of RHOB, RHOC, SIGLEC10 and GRK3 were not upregulated in CD14+ monocytes in SSc‐ILD lungs compared to controls compared to CD14+ monocytes in pseudobulk data (Figure 5B).
**Figure S16.** A) Natural killer (NK) cells genes (NCR3, RHD, MT‐CO2) did not show differences between disease groups in pseudobulk gene expression. B) CD4+ naïve T cells show trend toward increased pseudobulk gene expression of CHI3L2 from progressive SSc‐ILD while MT‐CO2 was significantly decreased compared to control. Horizontal line in scatterplot denotes the average.
**Figure S17.** RHD, NCR3, MT‐CO2, and CHI3L2 gene expression from different lung cell‐types.

**Table S1.** Upregualted gene expression by PBMC comparing progressive to stable disease ranked by p‐value*
**Table S2.** Upregulated gene expression by PBMC ranked by fold‐change progressive to stable disease, p‐value
**Table S3.** Gene Ontology biological pathways for genes upregulated in progressive PBMC (p,0.05)
**Table S4.** Downregualted gene expression by PBMC comparing progressive to stable disease
**Table S5.** Differentialy expressed genes in cell populations comparing progressive to stable SSc‐ILD
