# Supplementary material for: Monocyte LOXHD1 and RHOB Expression Predictive of Progressive Systemic Sclerosis–Associated Interstitial Lung Disease
Source: Arthritis Care Res (Hoboken). 2026 Jan 29;78(4):456–68. doi: 10.1002/acr.25619 (PMC13034103; doi:10.1002/acr.25619)
Supplement: Supplementary file 2 — Data S1 Supporting Information [file ACR-78-456-s004.docx]

METHODS

*Study Cohort*

Blood samples were obtained from SSc patients in the University of Pittsburgh Dorothy P and Richard P Simmons Center for Interstitial Lung Disease or Falk Rheumatology Clinic, who completed baseline evaluations and consented to experimental studies using their venous phlebotomy samples. Complete descriptions of subject recruitment and clinical evaluations were previously detailed (9, 10). Spirometry and diffusing capacity were performed using ATS standards and standard reference equations (11-13) . This study was approved by the Institutional Review Board for Human Subject Research at the University of Pittsburgh (STUDY20030223). All subjects provided written informed consent (in accordance with the Declaration of Helsinki) prior to their participation in this study.

Archival PBMC samples and matched to date sera from the University of Pittsburgh Interstitial Lung Disease Simmons Center, and prospectively collected blood samples from patients seen in clinic were studied. Samples collected at baseline (enrollment blood collection) from patients with SSc-ILD were stratified into progressive disease based on one of five criteria: 1. Death within 2 years of enrollment from any cause; Lung Transplant within 2 years after enrollment; 3. FVC< 50% at time of or within 2 years after enrollment. 4. Greater than 10% decline in FVC over two years comparing both before and after blood collection; or 5. Initiated on new immunosuppressive medication for ILD within 3 months of blood collection. Patients not meeting one of these criteria were considered to have stable disease at the time of enrollment. Patients in whom criteria were ambiguous were adjudicated as stable or progressive by chart review by Drs. Lafyatis and Kass. Demographic information and autoantibody reactivities were recorded for each patient, as available from the medical chart. Figure 1A shows how both prospective and archival samples were utilized.

*Serum biomarker analyses*

CRP, KL-6 and SP-D levels were analyzed using citrate plasma collected at the same time as PBMC from each patient. CRP and SP-D were analyzed using Quantikine ELISA kits (DCRP00 and DSFPD0, R & D Systems, Minneapolis, MN). KL-6 levels were analyzed using Luminex kit (LXSAHM-01, R & D Systems) and Bioplex 200 (Bio-Rad Labs, Hercules, CA). Sera collected at the same time as PBMC from each patient were used for analyzing CPK levels through the UPMC central laboratory using an IFCC-CK (NAC) kit on Beckman Coulter AU680 and AU5800 analyzers. Two sided Mann-Whitney U test performed to determine statistical significance.

*RNA isolation*

For the historical samples, PBMC were isolated using citrate CPT vacutainer tubes (Beckman-Dickerson, Franklin Lakes, NJ) and stored in either Trizol or RNAlater (Thermo-Fisher) at -80^o^C until RNA isolation. Samples stored in RNAlater were lysed in Trizol during RNA isolation procedure. RNA isolation was followed standard protocol using Qiagen Rneasy mini kit (Qiagen, Caldwell, NJ). All RNA samples were quantified using Agilent RNA 6000 Nano Kit and Bioanalyzer (Agilent 2100, Santa Clara, CA). All samples passed quality control with integrity number >8.

*RNA Sequencing Library Generation and Sequencing*

RNA was assessed for quality (Agilent TapeStation 4150) and RNA concentration quantified (Qubit FLEX fluorometer). Libraries were generated with the Illumina Stranded mRNA Library Prep kit (catalog #20040534) according to the manufacturer’s instructions. Briefly, 100 ng of input RNA was used for each sample. Following adapter ligation, 13 cycles of indexing PCR were completed, using IDT for Illumina RNA UD Indexes (Illumina, catalog #20040553-6). Libraries were quality and quantity and assessed (Qubit FLEX fluorometer and Agilent TapeStation 4150). Libraries were normalized and pooled to 10 nM by calculating the concentration based off the fragment size (base pairs) and the concentration (ng/μl) of the libraries. Sequencing was performed on an Illumina NovaSeq 6000 (UPMC Genome Center). Libraries were sequenced on an S2 flow cell with a target of ~50 million reads per sample.

*Analysis of bulk RNA-seq data*

PBMC RNA gene expression was analyzed by RNA-sequencing (RNA-seq). We then used three approaches to examine the RNA-seq data: Examining genes that were differentially expressed between stable and progressive patients using DESeq2, examining pathways regulated by these genes with uncorrected p<0.05 using Gene Ontology (GO). False Discovery Rate (FDR) <5% was considered statistically significant. Genes were then clustered meeting an expression threshold for detection using Cluster 3.0 and Java TreeView. The uncorrected p-value was used to identify potential candidate genes.

*Single cell RNA-sequencing of PBMC.*

PBMC were collected from patients and healthy control subjects and stored frozen in 10% DMSO. Frozen PBMC were thawed in batches of 10 samples, including 2 healthy controls and 3 or 4 each of progressive and stable SSc-ILD samples. Samples were labeled with barcoded antibodies for multiplexing samples ((14), 10X Genomics), as described previously (15). Targeting 5,000 cells sample, cells were partitioned, and single cell libraries prepared using the V3 3’ chemistry (10X Genomics), according to the 10X Genomics protocol. Libraries were sequenced, and sequences demultiplexed and aligned in Cell Ranger (10X Genomics).

*Analysis of single cell RNA-seq data*

Cell gene expression matrices were generated from the aligned sequence data. The MAST statistical algorithm feature with adjusted p-value <0.05 in Seurat version 4 was used to discover differentially expressed genes in the single cell RNA-seq (scRNA-seq) data comparing cells from patients with progressive to cells from patients with stable SSc-ILD cluster by cluster. Cells were plotted by transcriptome using the Uniform Manifold Approximation and Projection (UMAP) algorithm for dimensionality reduction, annotated using Azimuth (15) (Figure 3A), and confirmed cluster identities using known marker genes (Figure S8). We used Kruskal-Wallis test to compare control, stable, and progressive disease. Dunn’s test was performed following Kruskal-Wallis to identify which specific groups differ.
