# Supplementary material for: Monocyte LOXHD1 and RHOB Expression Predictive of Progressive Systemic Sclerosis–Associated Interstitial Lung Disease
Source: Arthritis Care Res (Hoboken). 2026 Jan 29;78(4):456–68. doi: 10.1002/acr.25619 (PMC13034103; doi:10.1002/acr.25619)
Supplement: Supplementary file 3 — Figure S1. Differential expressed genes of monocytes by single cell RNA‐seq of S100A12, S100A9, FPR2, and LOXHD1 between SSc‐ILD and control (HC/NOR). Figure S2. Upregulation of MMP9 in SSc‐ILD SPP1 macrophages by single cell RNA‐seq. Increased MMP9 gene expression in progressive disease compared to stable (p‐value=0.049. Horizontal line in scatterplot denotes the average. Figure S3. T cell receptors (TRAV21, TRBV19) and IL10RA showed decreased expression in scRNA‐seq from scleroderma cohort. Figure S4. Full gene list for each cluster group. Figure S5. A cluster of Y chromosome genes and a cluster of hemoglobin and other genes associated with red blood cells from reticulocytes. They may be related to vascular changes observed in SSc (e.g. telangiectasia, gastric antral vascular ectasia). Figure S6. A) CHIT1 and CHI3L1 and their associated genes. Chitinase genes encode serum biomarkers of fibrotic lung disease and trended toward higher expression in SSc‐ILD. B) RHOB and SAT1 and their associated genes. Figure S7. A) Pseudobulk RNA expression of CHI3L1 between progressive and stable SSc‐ILD. B) Increased single cell RNA expression of CHIT1 and CHI3L1 in SSc‐ILD SPP1 macrophages compared to control (NOR). P‐value is uncorrected and horizontal line in scatterplot denotes the average. Figure S8. Feature Plots showing known gene markers to confirm identities of different clusters in the UMAP (Figure 3) Figure S9. EIF4E, PIK3CG, and PRELID1 gene expression in CD14+ monocytes between control, stable, and progressive SSc‐ILD. Horizontal line in scatterplot denotes the average. Figure S10. ATXN2L gene expression in different lung cell types between control and SSc. Figure S11. RELB and NFKB1 showing decreased expression in SSc‐ILD CD14+ monocytes (by sample FDR<0.05) Figure S12. NFKB1 is decreased in multiple cell populations in SSc‐ILD lungs compared to controls. Figure S13. OSM, CASS4, PRDM1, RAPGEF1 show decreased expression in SSc‐ILD CD14+ monocytes (by sample FDR<0.05) [file ACR-78-456-s003.pdf]

Differentially Expressed Genes of Monocytes by Single cell RNA-seq between SSc-ILD and Control (HC)

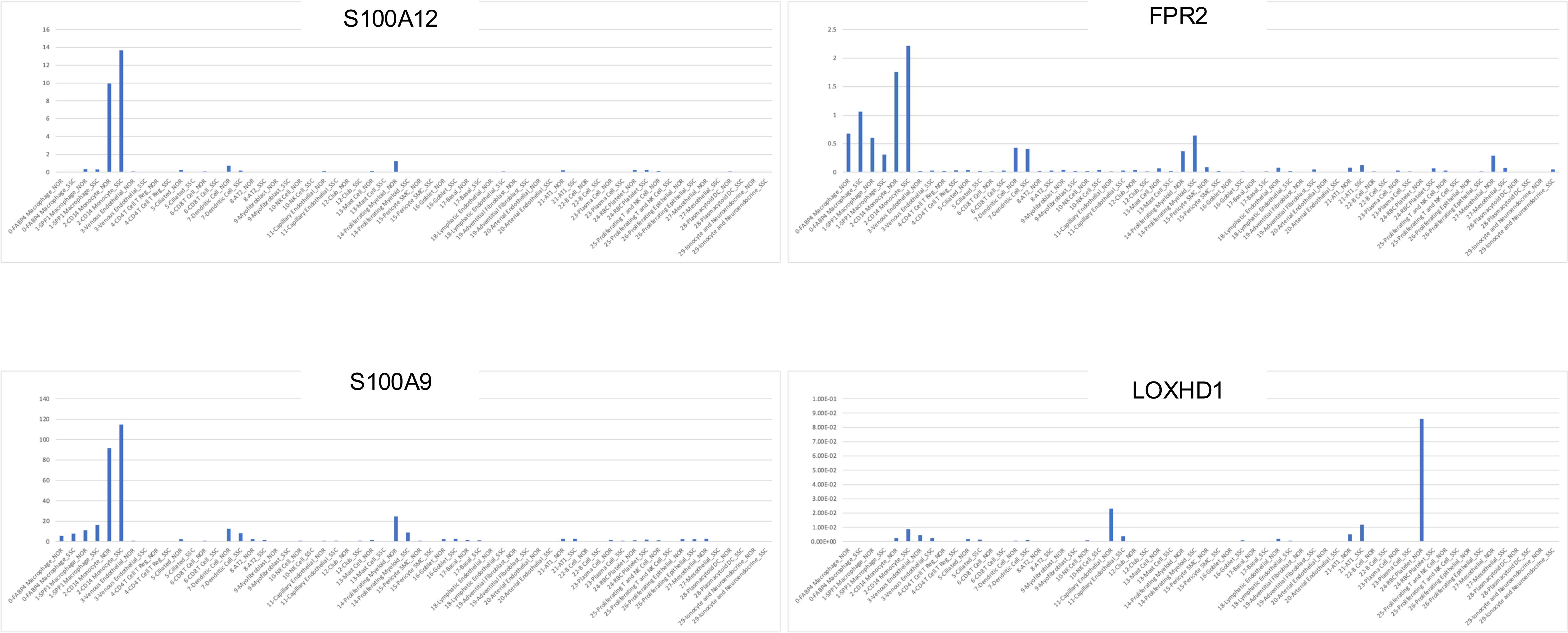

Figure S1

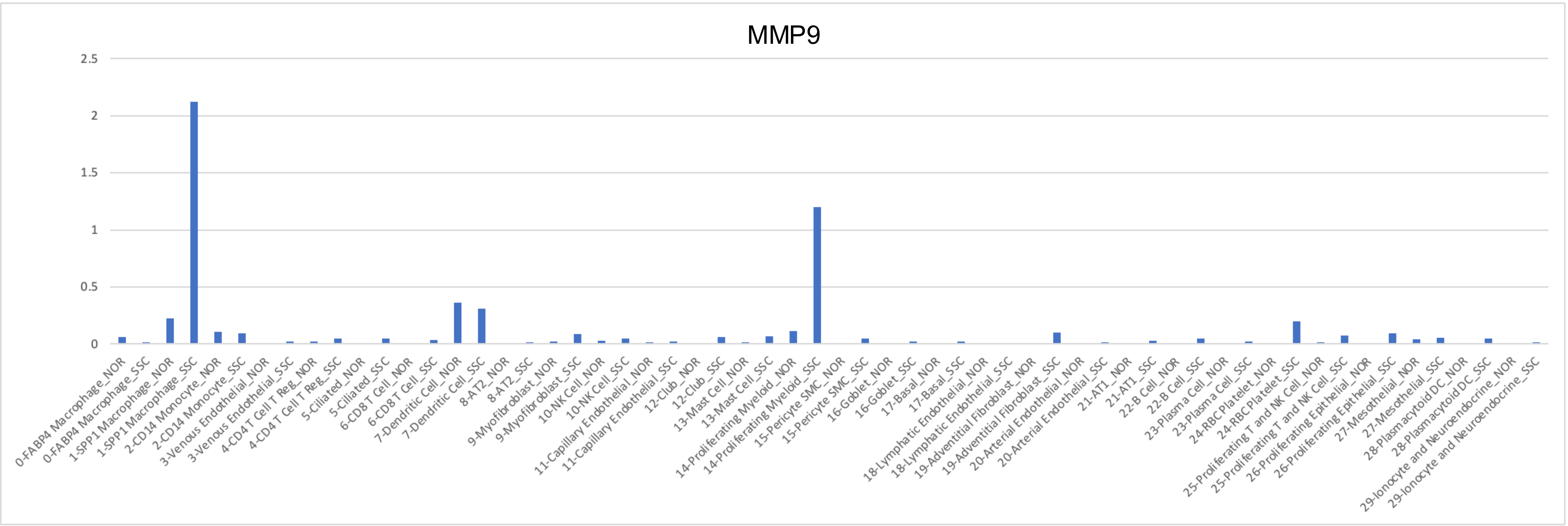
MMP9
2.5
2
1.5
1
0.5
0
0-FABP4 Macrophage_NOR
0-FABP4 Macrophage_SSC
1-SPP1 Macrophage_NOR
1-SPP1 Macrophage_SSC
2-CD14 Monocyte_NOR
2-CD14 Monocyte_SSC
3-Venous Endothelial_NOR
3-Venous Endothelial_SSC
4-CD4T Cell T Reg_NOR
4-CD4T Cell T Reg_SSC
5-Ciliated_NOR
5-Ciliated_SSC
6-CD8T Cell_NOR
6-CD8T Cell_SSC
7-Dendritic Cell_NOR
7-Dendritic Cell_SSC
8-AT2_NOR
8-AT2_SSC
9-Myofibroblast_NOR
9-Myofibroblast_SSC
10-NK Cell_NOR
10-NK Cell_SSC
11-Capillary Endothelial_NOR
11-Capillary Endothelial_SSC
12-Club_NOR
12-Club_SSC
13-Mast Cell_NOR
13-Mast Cell_SSC
14-Proliferating Myeloid_NOR
14-Proliferating Myeloid_SSC
15-Pericyte SMC_NOR
15-Pericyte SMC_SSC
16-Goblet_NOR
16-Goblet_SSC
17-Basal_NOR
17-Basal_SSC
18-Lymphatic Endothelial_NOR
18-Lymphatic Endothelial_SSC
19-Adventitial Fibroblast_NOR
19-Adventitial Fibroblast_SSC
20-Arterial Endothelial_NOR
20-Arterial Endothelial_SSC
21-AT1_NOR
21-AT1_SSC
22-B Cell_NOR
22-B Cell_SSC
23-Plasma Cell_NOR
23-Plasma Cell_SSC
24-RBC Platelet_NOR
24-RBC Platelet_SSC
25-Proliferating T and NK Cell_NOR
25-Proliferating T and NK Cell_SSC
26-Proliferating Epithelial_NOR
26-Proliferating Epithelial_SSC
27-Mesothelial_NOR
27-Mesothelial_SSC
28-Plasmacytoid DC_NOR
28-Plasmacytoid DC_SSC
29-Ionocyte and Neuroendocrine_NOR
29-Ionocyte and Neuroendocrine_SSC

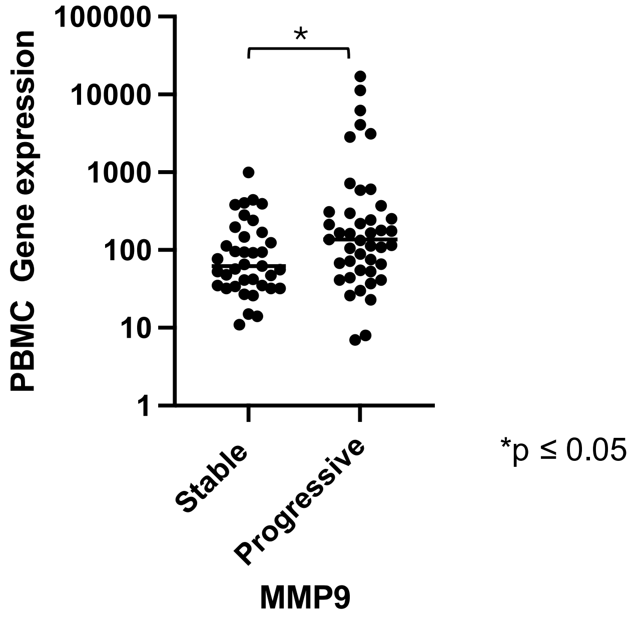
PBMC Gene expression
100000
10000
1000
100
10
1
*
Stable
Progressive
MMP9
*p ≤ 0.05

Figure S2

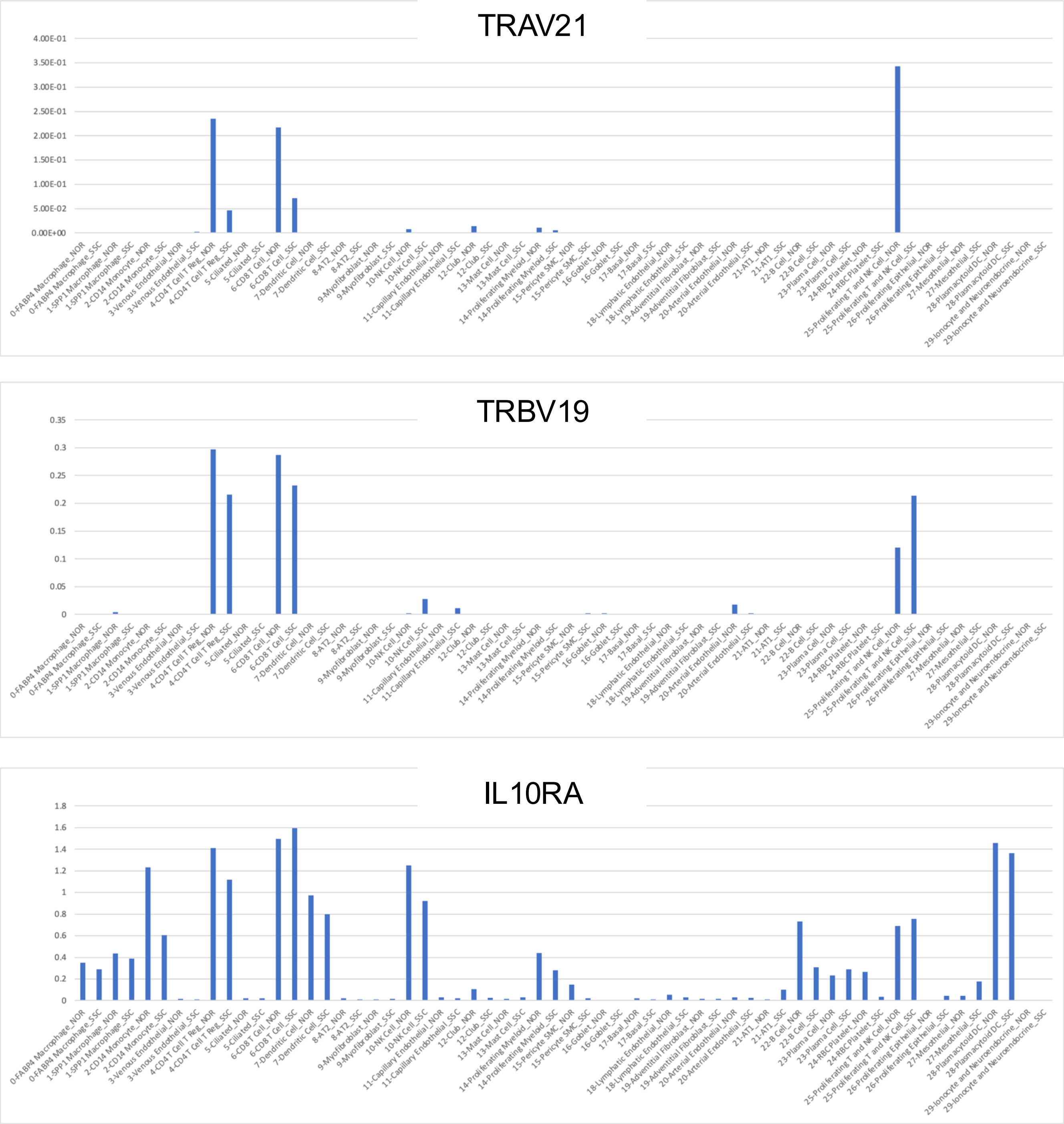
TRAV21
4.00E-01
3.50E-01
3.00E-01
2.50E-01
2.00E-01
1.50E-01
1.00E-01
5.00E-02
0.00E+00
TRBV19
0.35
0.3
0.25
0.2
0.15
0.1
0.05
0
IL10RA
1.8
1.6
1.4
1.2
1
0.8
0.6
0.4
0.2
0

Figure S3

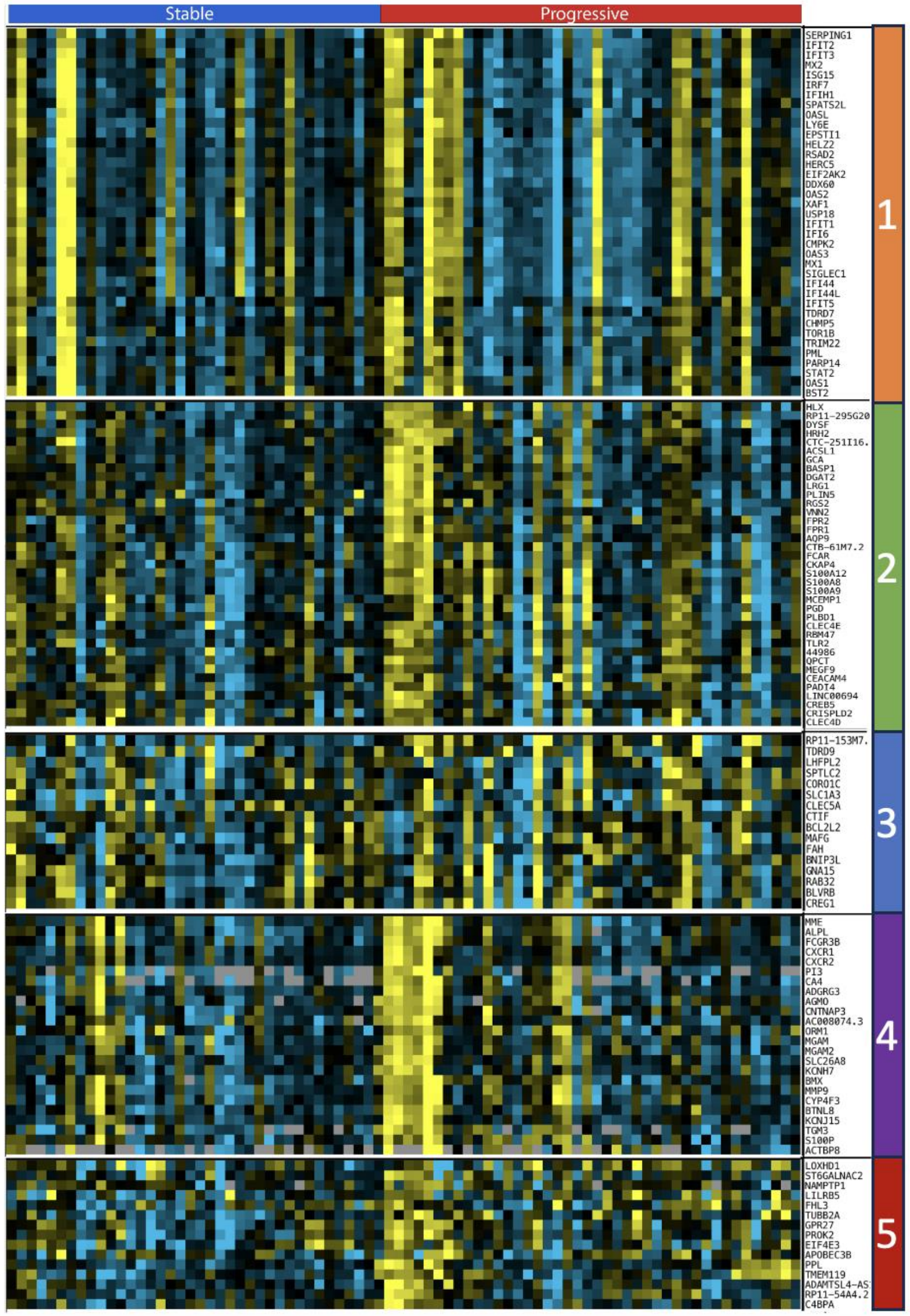
Stable
Progressive
SERPING1
IFIT2
IFIT3
MX2
ISG15
IRF7
IFIH1
SPATS2L
OASL
LY6E
EPSTI1
HELZ2
RSAD2
HERC5
EIF2AK2
DDX60
OAS2
XAF1
USP18
IFIT1
IFI6
CMPK2
OAS3
MX1
SIGLEC1
IFI44
IFI44L
IFIT5
TDRD7
CHMP5
TOR1B
TRIM22
PML
PARP14
STAT2
OAS1
BST2
HLX
RP11-295G20
DYSF
HRH2
CTC-251I16.
ACSL1
GCA
BASP1
DGAT2
LRG1
PLIN5
RGS2
VNN2
FPR2
FPR1
AQP9
CTB-61M7.2
FCAR
CKAP4
S100A12
S100A8
S100A9
MCEMP1
PGD
PLBD1
CLEC4E
RBM47
TLR2
44986
QPCT
MEGF9
CEACAM4
PADI4
LINC00694
CREB5
CRISPLD2
CLEC4D
RP11-153M7.
TDRD9
LHFPL2
SPTLC2
CORO1C
SLC1A3
CLEC5A
CTIF
BCL2L2
MAFG
FAH
BNIP3L
GNA15
RAB32
BLVRB
CREG1
MME
ALPL
FCGR3B
CXCR1
CXCR2
PI3
CA4
ADGRG3
AGMO
CNTNAP3
AC008074.3
ORM1
MGAM
MGAM2
SLC26A8
KCNH7
BMX
MMP9
CYP4F3
BTNL8
KCNJ15
TGM3
S100P
ACTBP8
LOXHD1
ST6GALNAC2
NAMPTP1
LILRB5
FHL3
TUBB2A
GPR27
PROK2
EIF4E3
APOBEC3B
PPL
TMEM119
ADAMTSL4-AS
RP11-54A4.2
C4BPA
1
2
3
4
5

Figure S4

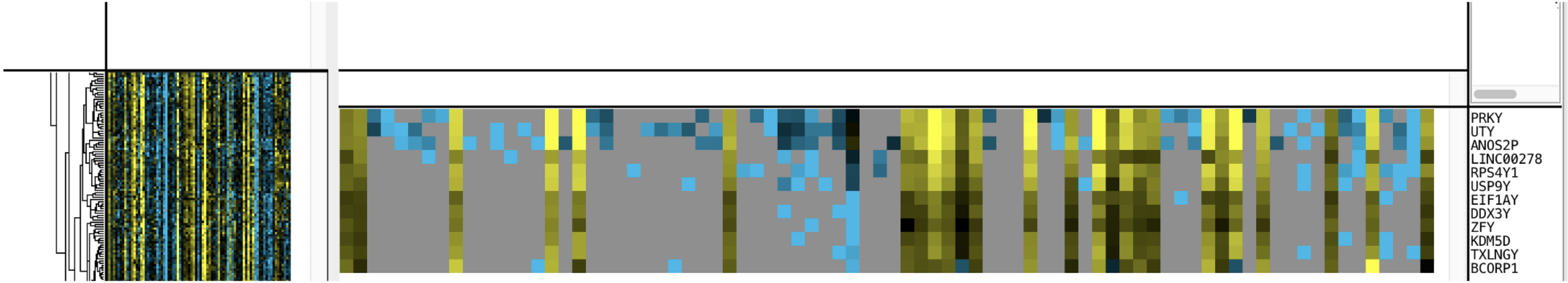

Male gene (Y) cluster

A

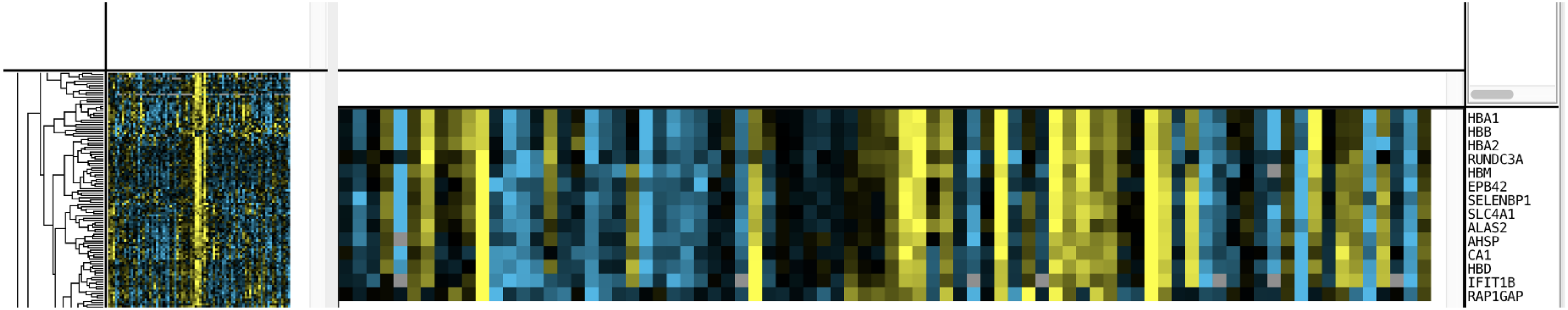

Reticulocyte gene cluster

B

FIGURE S5

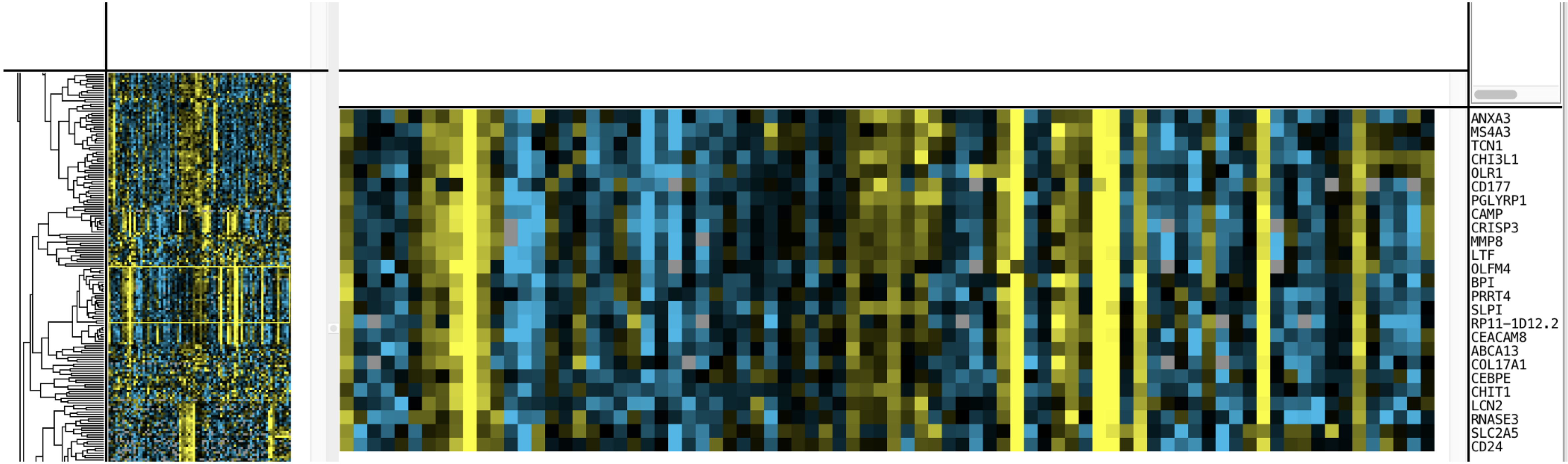

A CHIT1 and CHI3L1 gene cluster

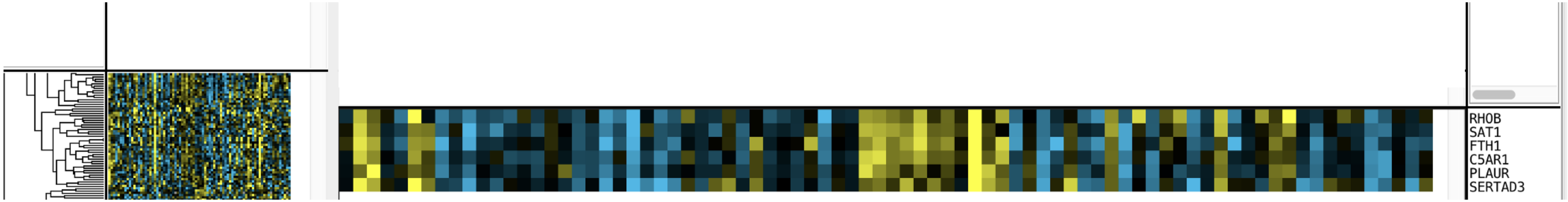

B RHOB and SAT1 gene cluster

FIGURE S6

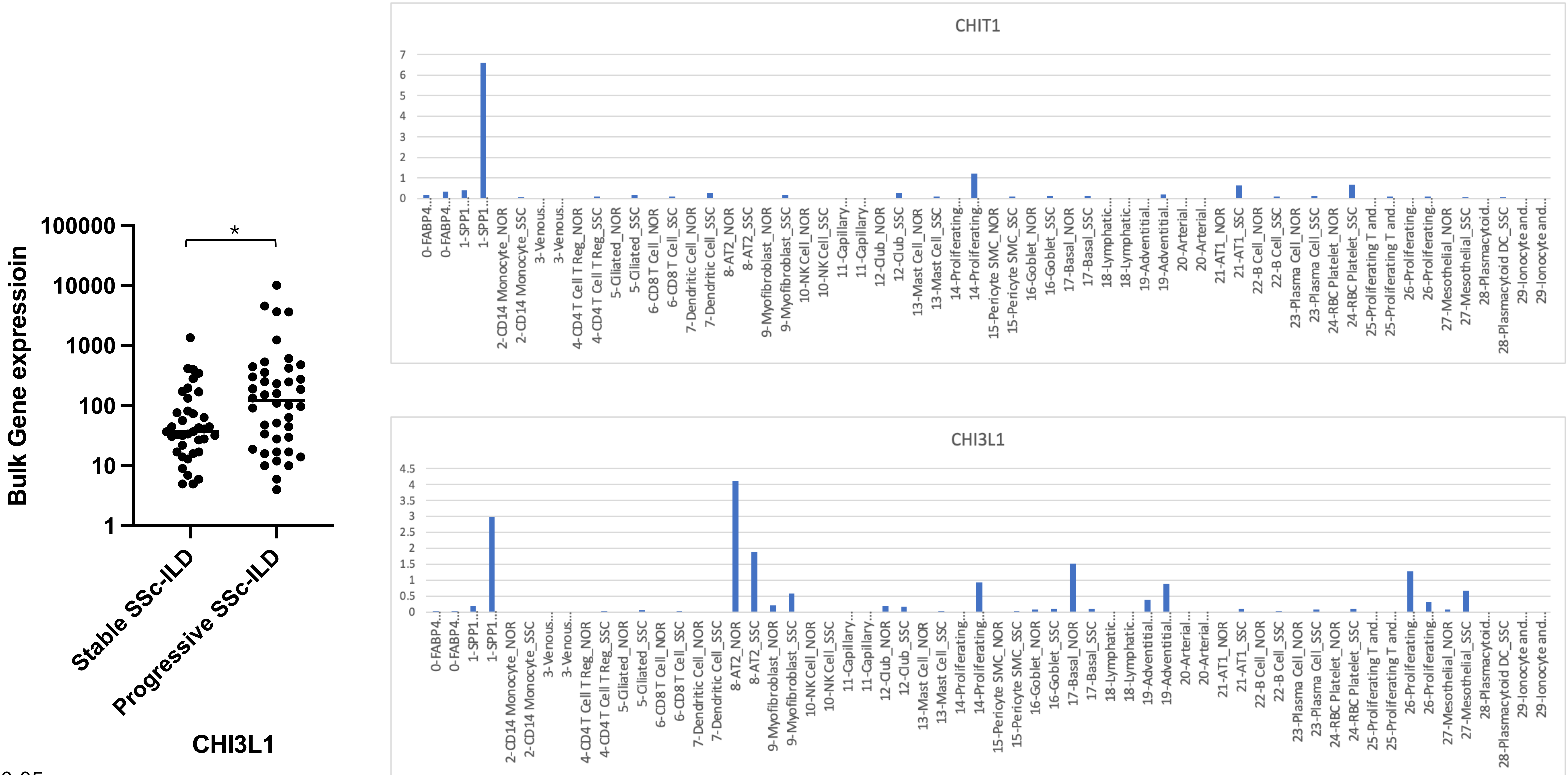

*p ≤ 0.05

A B

FIGURE S7

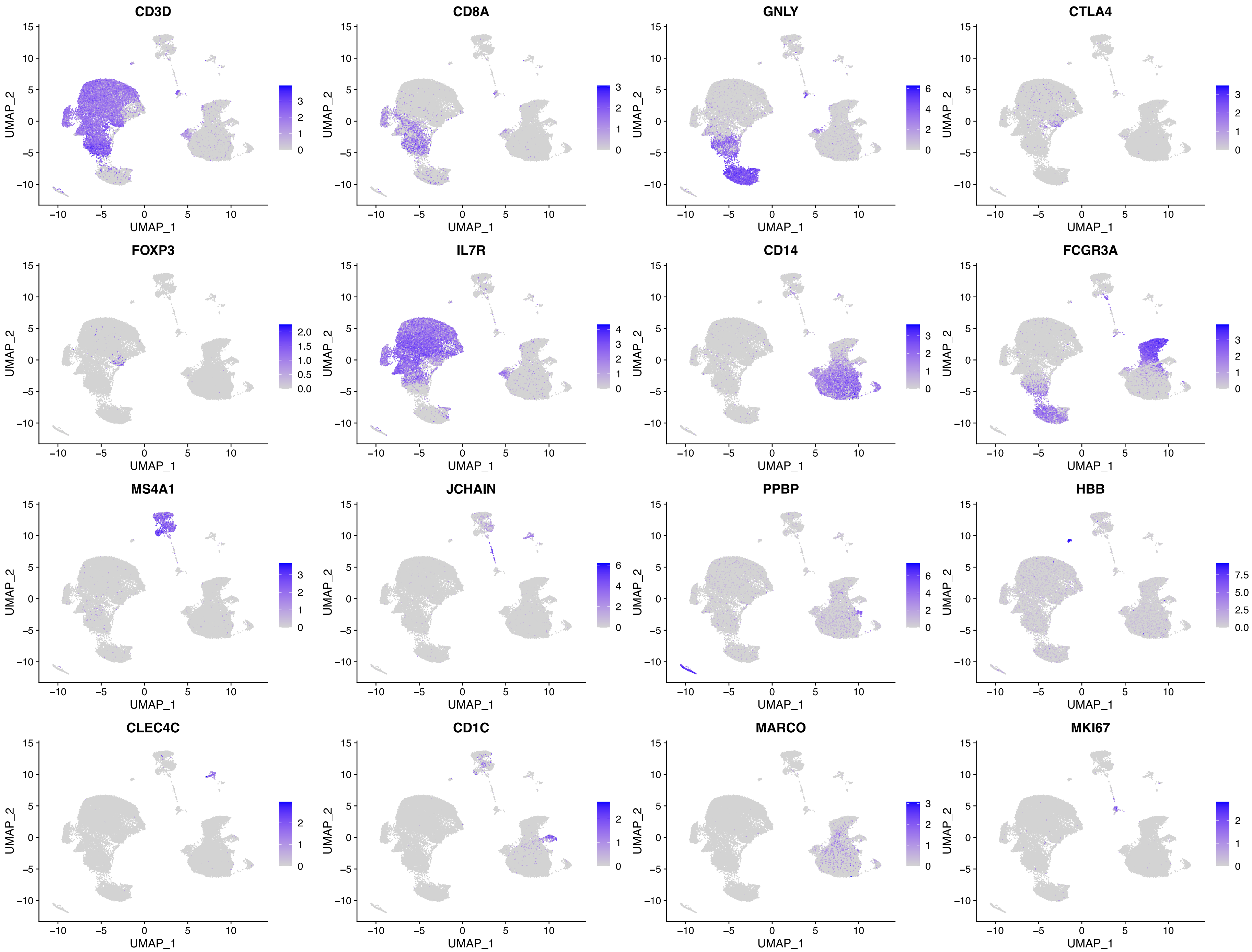
CD3D
CD8A
GNLY
CTLA4
FOXP3
IL7R
CD14
FCGR3A
MS4A1
JCHAIN
PPBP
HBB
CLEC4C
CD1C
MARCO
MKI67
UMAP_1
UMAP_2

Figure S8

CD14+ Monocytes

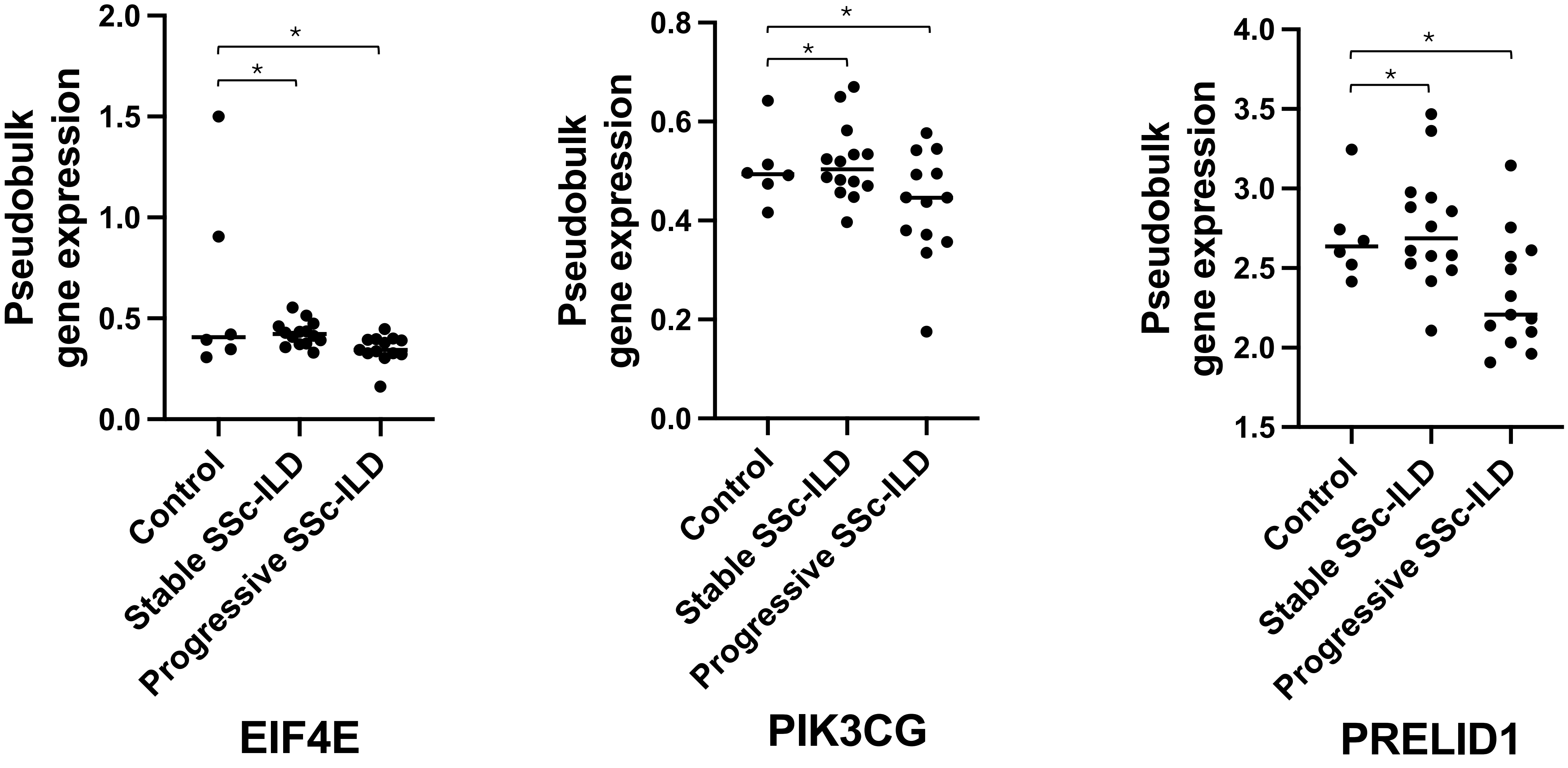

FIGURE S9

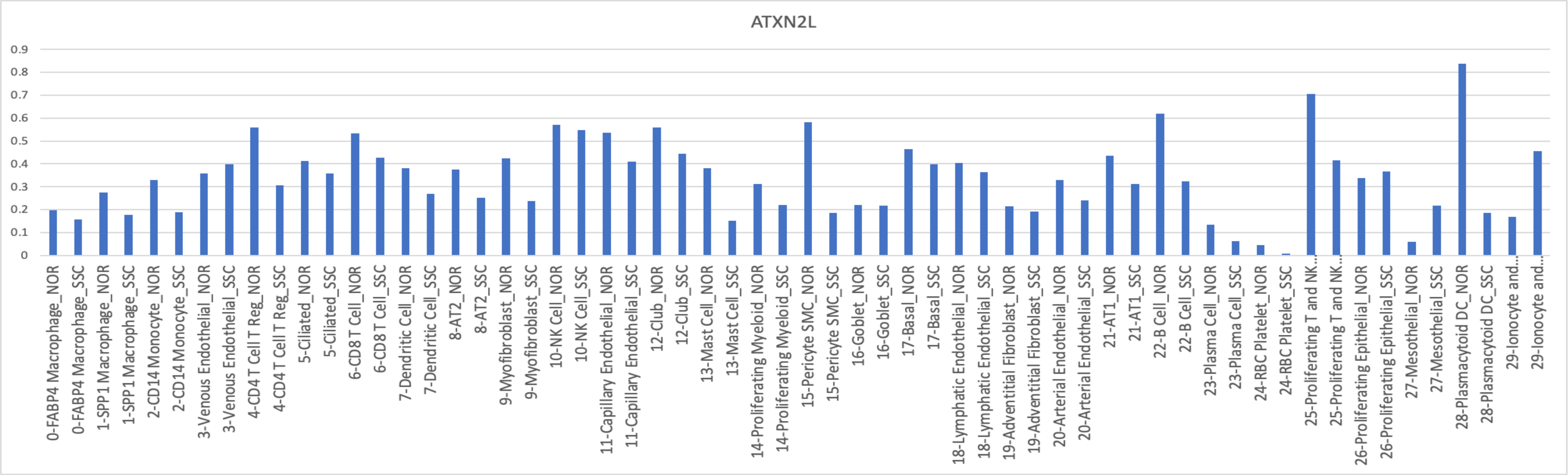
ATXN2L
0.9
0.8
0.7
0.6
0.5
0.4
0.3
0.2
0.1
0
0-FABP4 Macrophage_NOR
0-FABP4 Macrophage_SSC
1-SPP1 Macrophage_NOR
1-SPP1 Macrophage_SSC
2-CD14 Monocyte_NOR
2-CD14 Monocyte_SSC
3-Venous Endothelial_NOR
3-Venous Endothelial_SSC
4-CD4 T Cell T Reg_NOR
4-CD4 T Cell T Reg_SSC
5-Ciliated_NOR
5-Ciliated_SSC
6-CD8 T Cell_NOR
6-CD8 T Cell_SSC
7-Dendritic Cell_NOR
7-Dendritic Cell_SSC
8-AT2_NOR
8-AT2_SSC
9-Myofibroblast_NOR
9-Myofibroblast_SSC
10-NK Cell_NOR
10-NK Cell_SSC
11-Capillary Endothelial_NOR
11-Capillary Endothelial_SSC
12-Club_NOR
12-Club_SSC
13-Mast Cell_NOR
13-Mast Cell_SSC
14-Proliferating Myeloid_NOR
14-Proliferating Myeloid_SSC
15-Pericyte SMC_NOR
15-Pericyte SMC_SSC
16-Goblet_NOR
16-Goblet_SSC
17-Basal_NOR
17-Basal_SSC
18-Lymphatic Endothelial_NOR
18-Lymphatic Endothelial_SSC
19-Adventitial Fibroblast_NOR
19-Adventitial Fibroblast_SSC
20-Arterial Endothelial_NOR
20-Arterial Endothelial_SSC
21-AT1_NOR
21-AT1_SSC
22-B Cell_NOR
22-B Cell_SSC
23-Plasma Cell_NOR
23-Plasma Cell_SSC
24-RBC Platelet_NOR
24-RBC Platelet_SSC
25-Proliferating T and NK...
25-Proliferating T and NK...
26-Proliferating Epithelial_NOR
26-Proliferating Epithelial_SSC
27-Mesothelial_NOR
27-Mesothelial_SSC
28-Plasmacytoid DC_NOR
28-Plasmacytoid DC_SSC
29-Ionocyte and...
29-Ionocyte and...

Figure S10

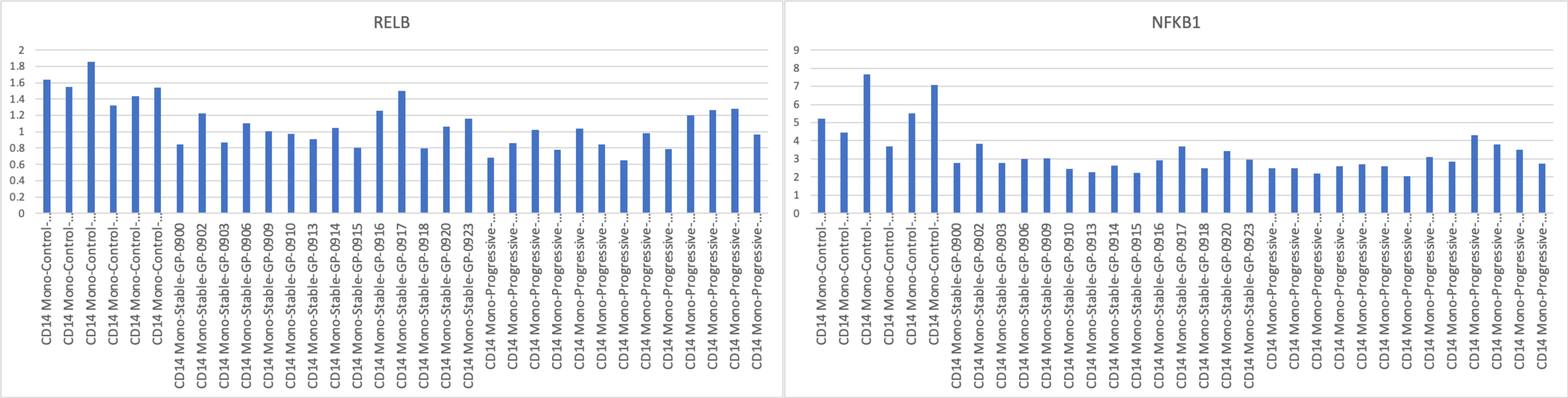
RELB
2
1.8
1.6
1.4
1.2
1
0.8
0.6
0.4
0.2
0
CD14 Mono-Control-...
CD14 Mono-Stable-GP-0900
CD14 Mono-Stable-GP-0902
CD14 Mono-Stable-GP-0903
CD14 Mono-Stable-GP-0906
CD14 Mono-Stable-GP-0909
CD14 Mono-Stable-GP-0910
CD14 Mono-Stable-GP-0913
CD14 Mono-Stable-GP-0914
CD14 Mono-Stable-GP-0915
CD14 Mono-Stable-GP-0916
CD14 Mono-Stable-GP-0917
CD14 Mono-Stable-GP-0918
CD14 Mono-Stable-GP-0920
CD14 Mono-Stable-GP-0923
CD14 Mono-Progressive-...
NFKB1
9
8
7
6
5
4
3
2
1
0
CD14 Mono-Control-...
CD14 Mono-Stable-GP-0900
CD14 Mono-Stable-GP-0902
CD14 Mono-Stable-GP-0903
CD14 Mono-Stable-GP-0906
CD14 Mono-Stable-GP-0909
CD14 Mono-Stable-GP-0910
CD14 Mono-Stable-GP-0913
CD14 Mono-Stable-GP-0914
CD14 Mono-Stable-GP-0915
CD14 Mono-Stable-GP-0916
CD14 Mono-Stable-GP-0917
CD14 Mono-Stable-GP-0918
CD14 Mono-Stable-GP-0920
CD14 Mono-Stable-GP-0923
CD14 Mono-Progressive-...

Figure S11

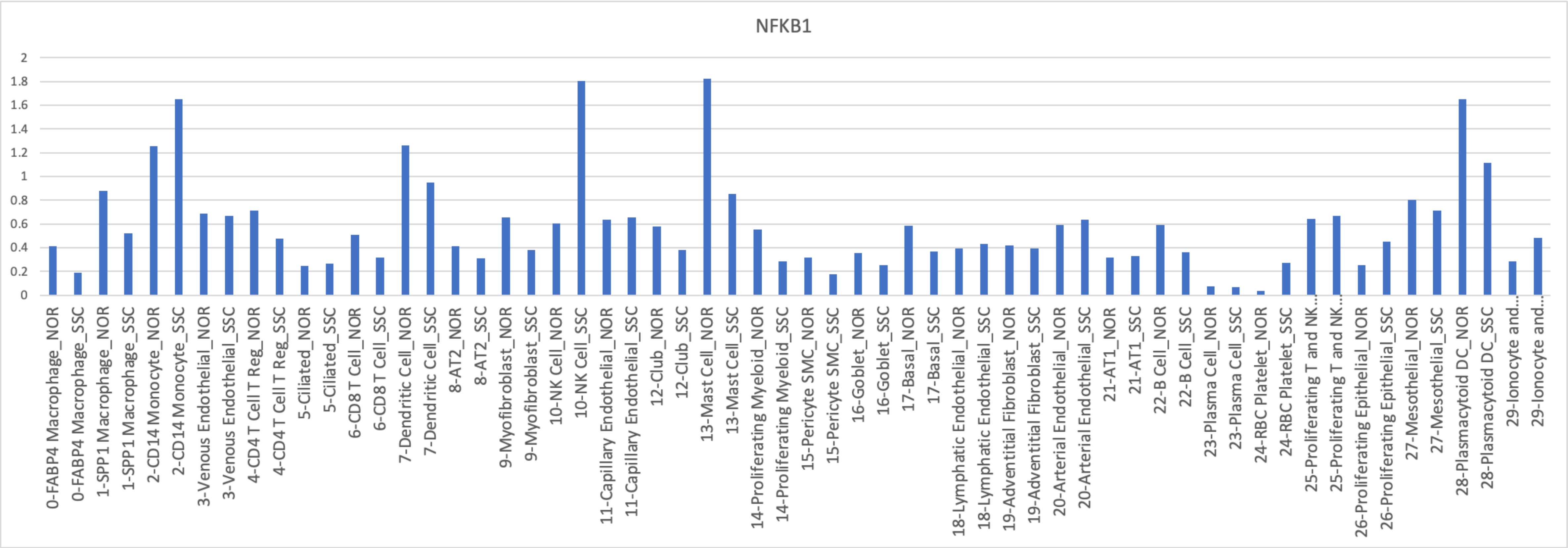
NFKB1
2
1.8
1.6
1.4
1.2
1
0.8
0.6
0.4
0.2
0
0-FABP4 Macrophage_NOR
0-FABP4 Macrophage_SSC
1-SPP1 Macrophage_NOR
1-SPP1 Macrophage_SSC
2-CD14 Monocyte_NOR
2-CD14 Monocyte_SSC
3-Venous Endothelial_NOR
3-Venous Endothelial_SSC
4-CD4 T Cell T Reg_NOR
4-CD4 T Cell T Reg_SSC
5-Ciliated_NOR
5-Ciliated_SSC
6-CD8 T Cell_NOR
6-CD8 T Cell_SSC
7-Dendritic Cell_NOR
7-Dendritic Cell_SSC
8-AT2_NOR
8-AT2_SSC
9-Myofibroblast_NOR
9-Myofibroblast_SSC
10-NK Cell_NOR
10-NK Cell_SSC
11-Capillary Endothelial_NOR
11-Capillary Endothelial_SSC
12-Club_NOR
12-Club_SSC
13-Mast Cell_NOR
13-Mast Cell_SSC
14-Proliferating Myeloid_NOR
14-Proliferating Myeloid_SSC
15-Pericyte SMC_NOR
15-Pericyte SMC_SSC
16-Goblet_NOR
16-Goblet_SSC
17-Basal_NOR
17-Basal_SSC
18-Lymphatic Endothelial_NOR
18-Lymphatic Endothelial_SSC
19-Adventitial Fibroblast_NOR
19-Adventitial Fibroblast_SSC
20-Arterial Endothelial_NOR
20-Arterial Endothelial_SSC
21-AT1_NOR
21-AT1_SSC
22-B Cell_NOR
22-B Cell_SSC
23-Plasma Cell_NOR
23-Plasma Cell_SSC
24-RBC Platelet_NOR
24-RBC Platelet_SSC
25-Proliferating T and NK...
25-Proliferating T and NK...
26-Proliferating Epithelial_NOR
26-Proliferating Epithelial_SSC
27-Mesothelial_NOR
27-Mesothelial_SSC
28-Plasmacytoid DC_NOR
28-Plasmacytoid DC_SSC
29-Ionocyte and...
29-Ionocyte and...

Figure S12

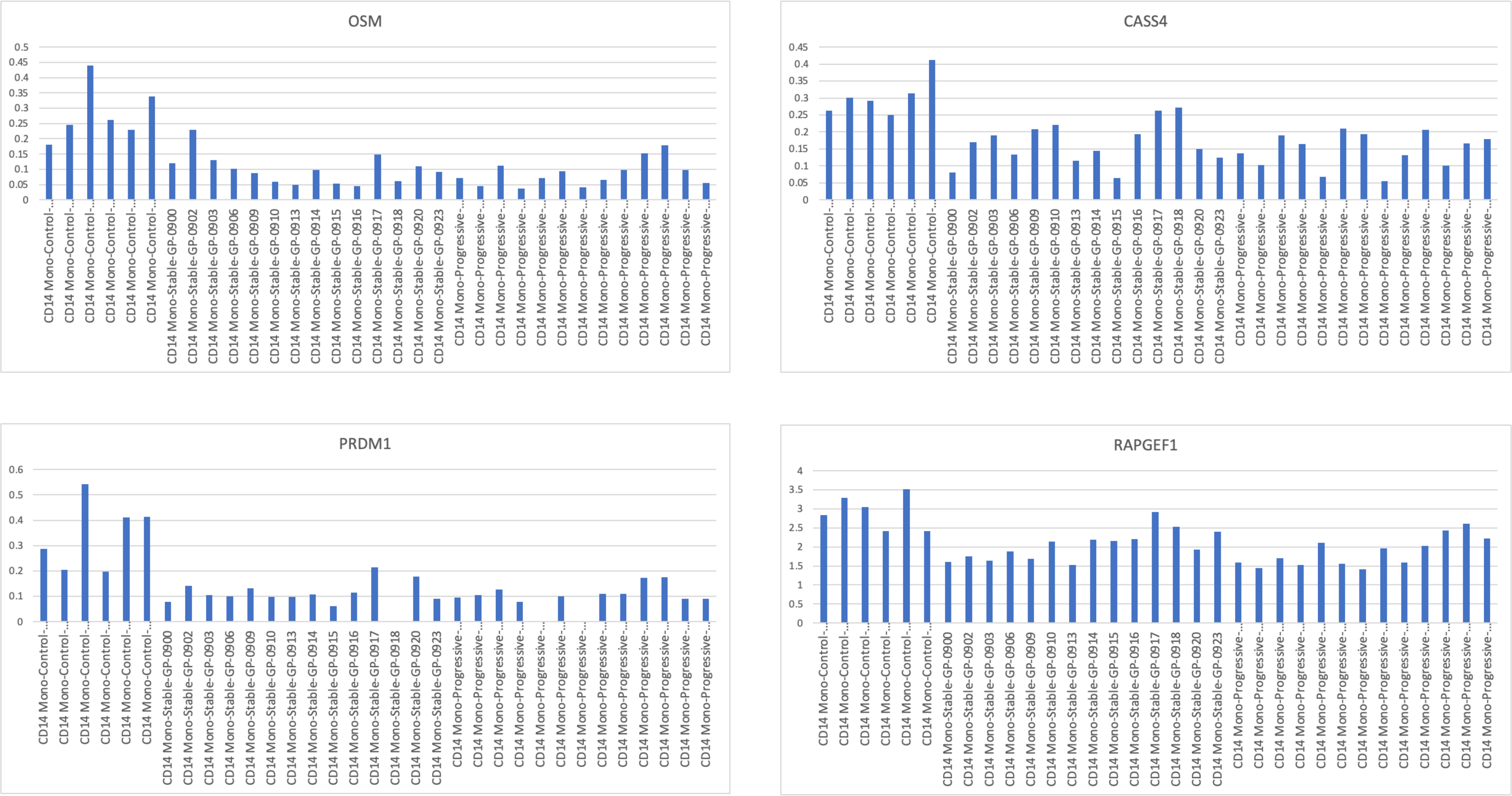
OSM
0.5
0.45
0.4
0.35
0.3
0.25
0.2
0.15
0.1
0.05
0
CD14 Mono-Control-...
CD14 Mono-Stable-GP-0900
CD14 Mono-Stable-GP-0902
CD14 Mono-Stable-GP-0903
CD14 Mono-Stable-GP-0906
CD14 Mono-Stable-GP-0909
CD14 Mono-Stable-GP-0910
CD14 Mono-Stable-GP-0913
CD14 Mono-Stable-GP-0914
CD14 Mono-Stable-GP-0915
CD14 Mono-Stable-GP-0916
CD14 Mono-Stable-GP-0917
CD14 Mono-Stable-GP-0918
CD14 Mono-Stable-GP-0920
CD14 Mono-Stable-GP-0923
CD14 Mono-Progressive-...
CASS4
0.45
0.4
0.35
0.3
0.25
0.2
0.15
0.1
0.05
0
PRDM1
0.6
0.5
0.4
0.3
0.2
0.1
0
RAPGEF1
4
3.5
3
2.5
2
1.5
1
0.5
0

Figure S13

CD16+ Monocytes

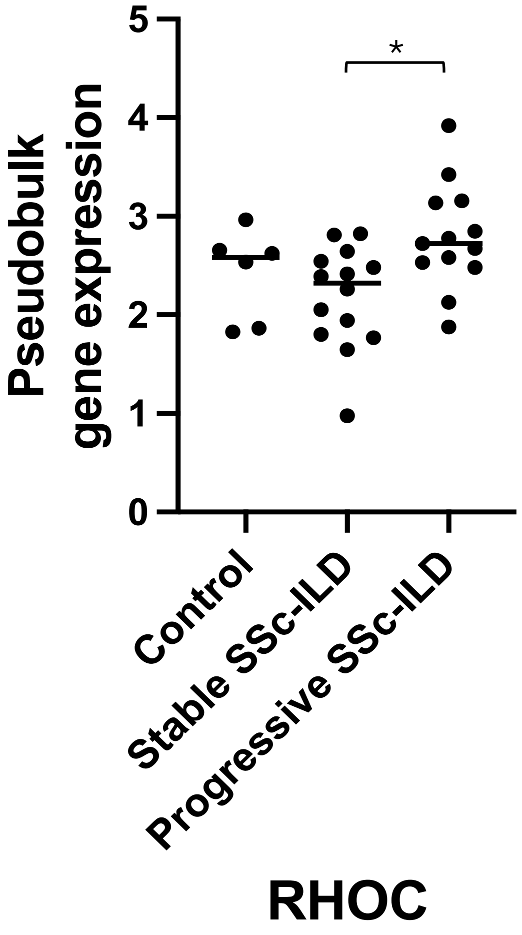

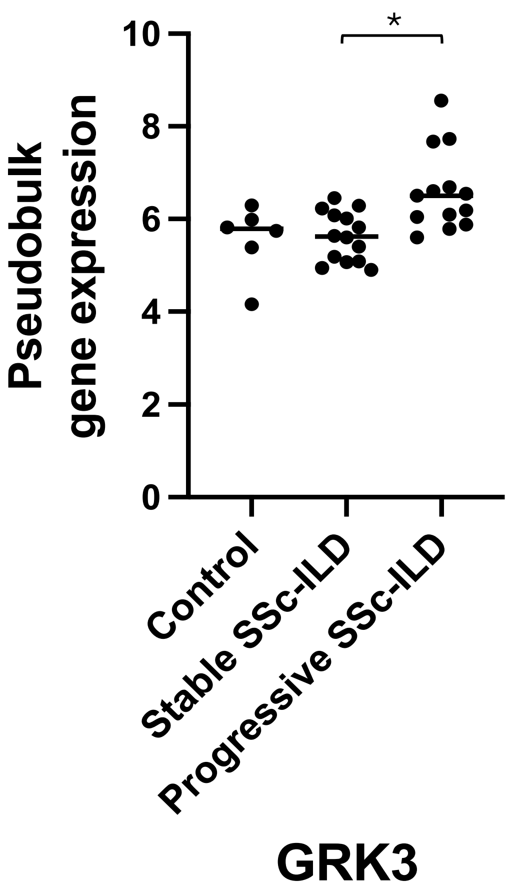

FIGURE S14

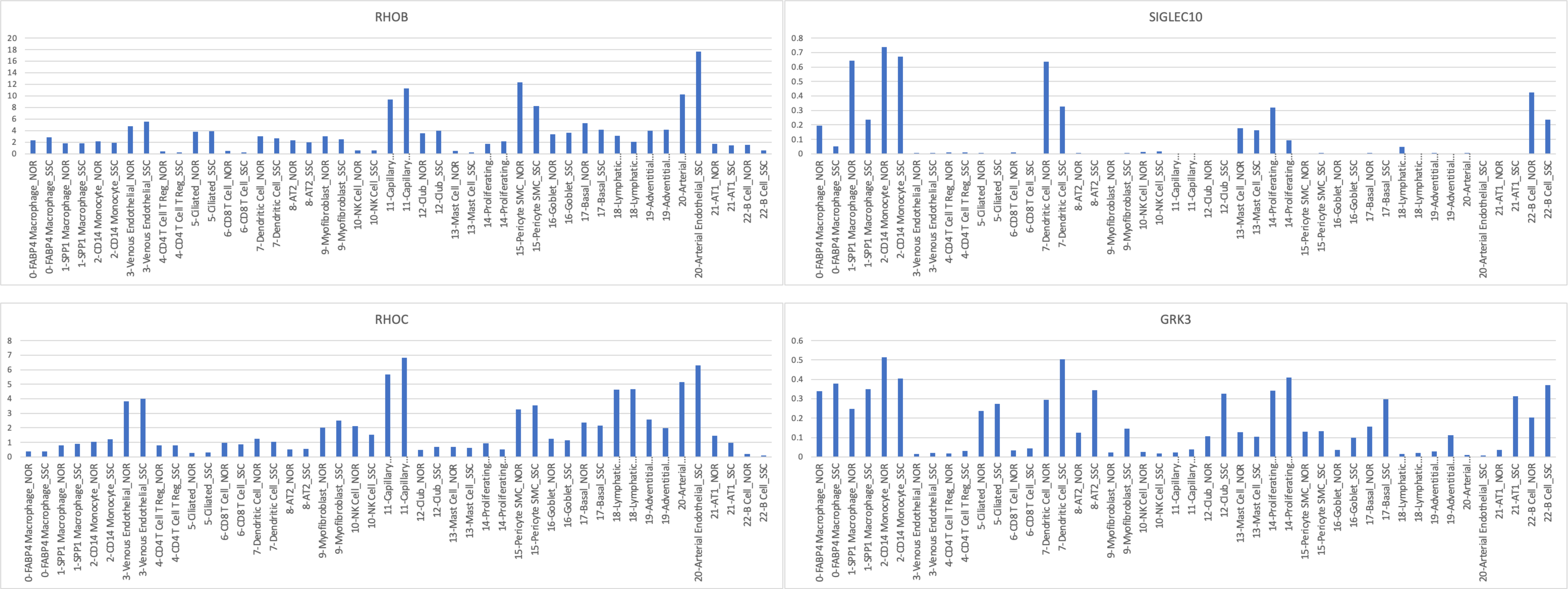
RHOB
20
18
16
14
12
10
8
6
4
2
0
0-FABP4 Macrophage_NOR
0-FABP4 Macrophage_SSC
1-SPP1 Macrophage_NOR
1-SPP1 Macrophage_SSC
2-CD14 Monocyte_NOR
2-CD14 Monocyte_SSC
3-Venous Endothelial_NOR
3-Venous Endothelial_SSC
4-CD4 T Cell T Reg_NOR
4-CD4 T Cell T Reg_SSC
5-Ciliated_NOR
5-Ciliated_SSC
6-CD8 T Cell_NOR
6-CD8 T Cell_SSC
7-Dendritic Cell_NOR
7-Dendritic Cell_SSC
8-AT2_NOR
8-AT2_SSC
9-Myofibroblast_NOR
9-Myofibroblast_SSC
10-NK Cell_NOR
10-NK Cell_SSC
11-Capillary...
11-Capillary...
12-Club_NOR
12-Club_SSC
13-Mast Cell_NOR
13-Mast Cell_SSC
14-Proliferating...
14-Proliferating...
15-Pericyte SMC_NOR
15-Pericyte SMC_SSC
16-Goblet_NOR
16-Goblet_SSC
17-Basal_NOR
17-Basal_SSC
18-Lymphatic...
18-Lymphatic...
19-Adventitial...
19-Adventitial...
20-Arterial...
20-Arterial Endothelial_SSC
21-AT1_NOR
21-AT1_SSC
22-B Cell_NOR
22-B Cell_SSC
SIGLEC10
0.8
0.7
0.6
0.5
0.4
0.3
0.2
0.1
0
0-FABP4 Macrophage_NOR
0-FABP4 Macrophage_SSC
1-SPP1 Macrophage_NOR
1-SPP1 Macrophage_SSC
2-CD14 Monocyte_NOR
2-CD14 Monocyte_SSC
3-Venous Endothelial_NOR
3-Venous Endothelial_SSC
4-CD4 T Cell T Reg_NOR
4-CD4 T Cell T Reg_SSC
5-Ciliated_NOR
5-Ciliated_SSC
6-CD8 T Cell_NOR
6-CD8 T Cell_SSC
7-Dendritic Cell_NOR
7-Dendritic Cell_SSC
8-AT2_NOR
8-AT2_SSC
9-Myofibroblast_NOR
9-Myofibroblast_SSC
10-NK Cell_NOR
10-NK Cell_SSC
11-Capillary...
11-Capillary...
12-Club_NOR
12-Club_SSC
13-Mast Cell_NOR
13-Mast Cell_SSC
14-Proliferating...
14-Proliferating...
15-Pericyte SMC_NOR
15-Pericyte SMC_SSC
16-Goblet_NOR
16-Goblet_SSC
17-Basal_NOR
17-Basal_SSC
18-Lymphatic...
18-Lymphatic...
19-Adventitial...
19-Adventitial...
20-Arterial...
20-Arterial Endothelial_SSC
21-AT1_NOR
21-AT1_SSC
22-B Cell_NOR
22-B Cell_SSC
RHOC
8
7
6
5
4
3
2
1
0
0-FABP4 Macrophage_NOR
0-FABP4 Macrophage_SSC
1-SPP1 Macrophage_NOR
1-SPP1 Macrophage_SSC
2-CD14 Monocyte_NOR
2-CD14 Monocyte_SSC
3-Venous Endothelial_NOR
3-Venous Endothelial_SSC
4-CD4 T Cell T Reg_NOR
4-CD4 T Cell T Reg_SSC
5-Ciliated_NOR
5-Ciliated_SSC
6-CD8 T Cell_NOR
6-CD8 T Cell_SSC
7-Dendritic Cell_NOR
7-Dendritic Cell_SSC
8-AT2_NOR
8-AT2_SSC
9-Myofibroblast_NOR
9-Myofibroblast_SSC
10-NK Cell_NOR
10-NK Cell_SSC
11-Capillary...
11-Capillary...
12-Club_NOR
12-Club_SSC
13-Mast Cell_NOR
13-Mast Cell_SSC
14-Proliferating...
14-Proliferating...
15-Pericyte SMC_NOR
15-Pericyte SMC_SSC
16-Goblet_NOR
16-Goblet_SSC
17-Basal_NOR
17-Basal_SSC
18-Lymphatic...
18-Lymphatic...
19-Adventitial...
19-Adventitial...
20-Arterial...
20-Arterial Endothelial_SSC
21-AT1_NOR
21-AT1_SSC
22-B Cell_NOR
22-B Cell_SSC
GRK3
0.6
0.5
0.4
0.3
0.2
0.1
0
0-FABP4 Macrophage_NOR
0-FABP4 Macrophage_SSC
1-SPP1 Macrophage_NOR
1-SPP1 Macrophage_SSC
2-CD14 Monocyte_NOR
2-CD14 Monocyte_SSC
3-Venous Endothelial_NOR
3-Venous Endothelial_SSC
4-CD4 T Cell T Reg_NOR
4-CD4 T Cell T Reg_SSC
5-Ciliated_NOR
5-Ciliated_SSC
6-CD8 T Cell_NOR
6-CD8 T Cell_SSC
7-Dendritic Cell_NOR
7-Dendritic Cell_SSC
8-AT2_NOR
8-AT2_SSC
9-Myofibroblast_NOR
9-Myofibroblast_SSC
10-NK Cell_NOR
10-NK Cell_SSC
11-Capillary...
11-Capillary...
12-Club_NOR
12-Club_SSC
13-Mast Cell_NOR
13-Mast Cell_SSC
14-Proliferating...
14-Proliferating...
15-Pericyte SMC_NOR
15-Pericyte SMC_SSC
16-Goblet_NOR
16-Goblet_SSC
17-Basal_NOR
17-Basal_SSC
18-Lymphatic...
18-Lymphatic...
19-Adventitial...
19-Adventitial...
20-Arterial...
20-Arterial Endothelial_SSC
21-AT1_NOR
21-AT1_SSC
22-B Cell_NOR
22-B Cell_SSC

Figure S15

CD4+ naïve T cells

NK cells

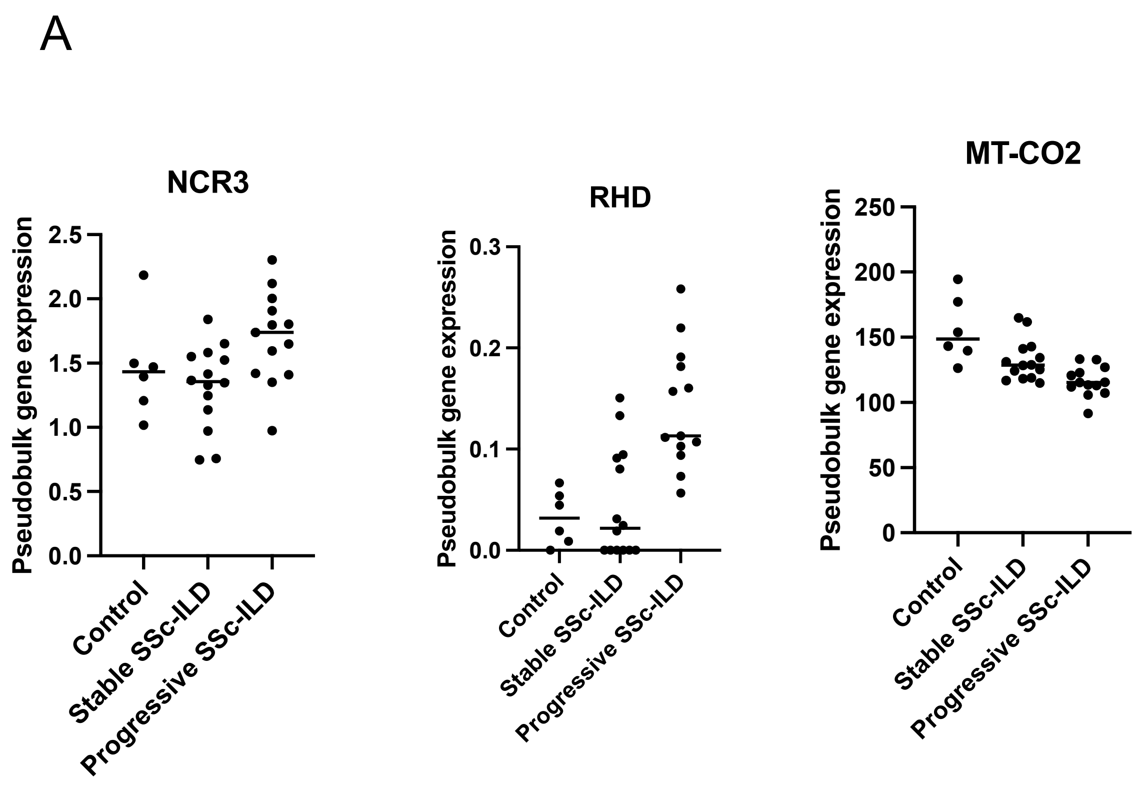

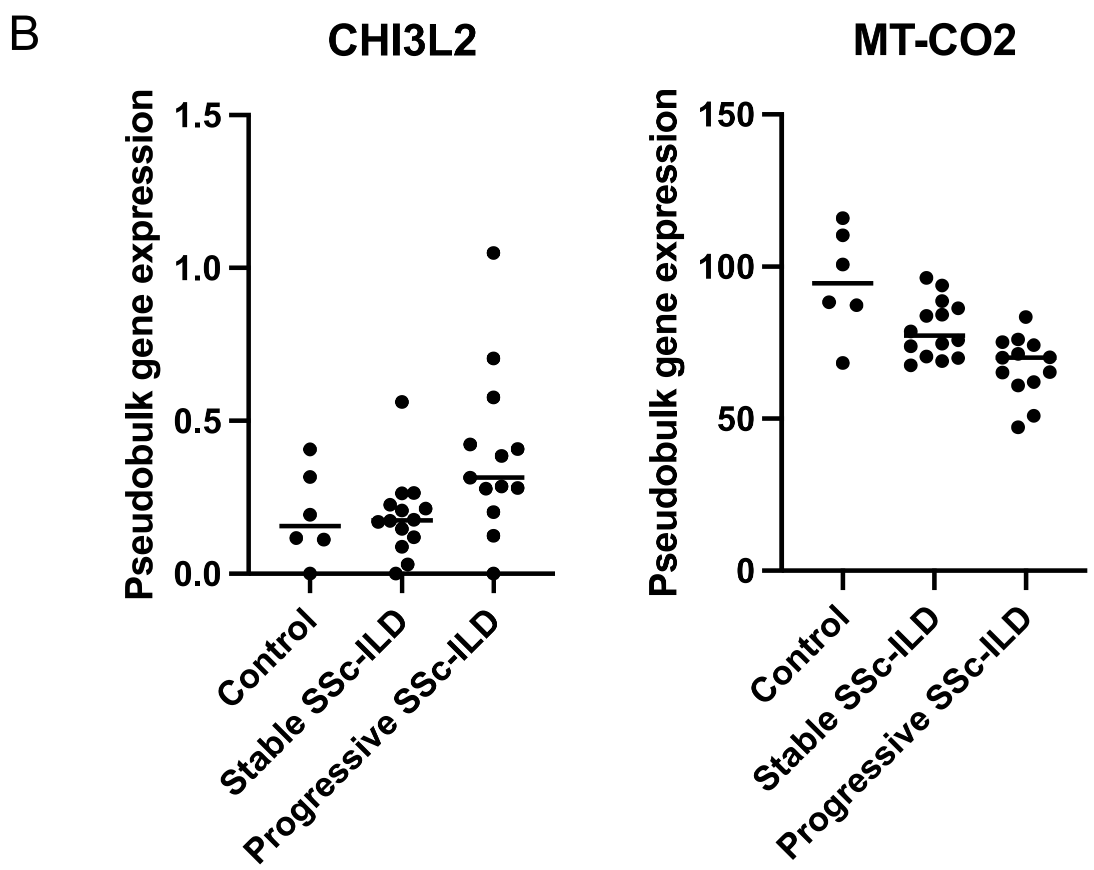

Figure S16

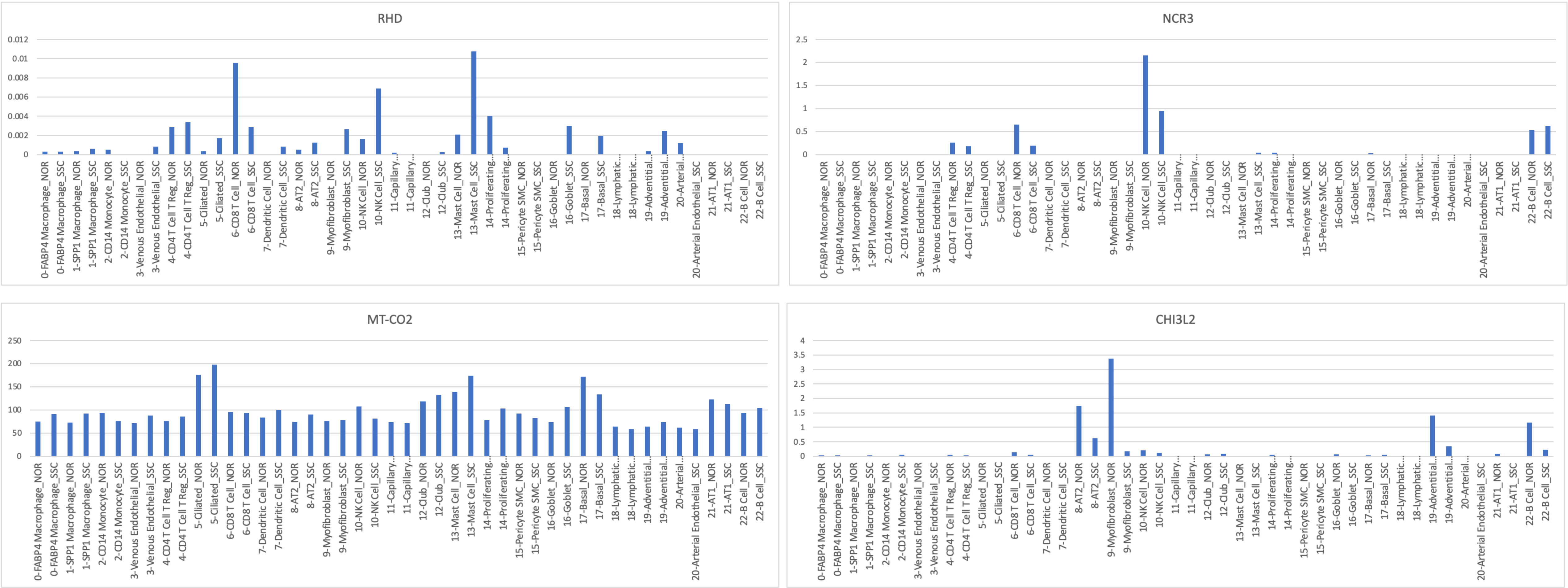
RHD
0.012
0.01
0.008
0.006
0.004
0.002
0
0-FABP4 Macrophage_NOR
0-FABP4 Macrophage_SSC
1-SPP1 Macrophage_NOR
1-SPP1 Macrophage_SSC
2-CD14 Monocyte_NOR
2-CD14 Monocyte_SSC
3-Venous Endothelial_NOR
3-Venous Endothelial_SSC
4-CD4T Cell T Reg_NOR
4-CD4 T Cell T Reg_SSC
5-Ciliated_NOR
5-Ciliated_SSC
6-CD8T Cell_NOR
6-CD8 T Cell_SSC
7-Dendritic Cell_NOR
7-Dendritic Cell_SSC
8-AT2_NOR
8-AT2_SSC
9-Myofibroblast_NOR
9-Myofibroblast_SSC
10-NK Cell_NOR
10-NK Cell_SSC
11-Capillary...
11-Capillary...
12-Club_NOR
12-Club_SSC
13-Mast Cell_NOR
13-Mast Cell_SSC
14-Proliferating...
14-Proliferating...
15-Pericyte SMC_NOR
15-Pericyte SMC_SSC
16-Goblet_NOR
16-Goblet_SSC
17-Basal_NOR
17-Basal_SSC
18-Lymphatic...
18-Lymphatic...
19-Adventitial...
19-Adventitial...
20-Arterial...
20-Arterial Endothelial_SSC
21-AT1_NOR
21-AT1_SSC
22-B Cell_NOR
22-B Cell_SSC
NCR3
2.5
2
1.5
1
0.5
0
0-FABP4 Macrophage_NOR
0-FABP4 Macrophage_SSC
1-SPP1 Macrophage_NOR
1-SPP1 Macrophage_SSC
2-CD14 Monocyte_NOR
2-CD14 Monocyte_SSC
3-Venous Endothelial_NOR
3-Venous Endothelial_SSC
4-CD4T Cell T Reg_NOR
4-CD4 T Cell T Reg_SSC
5-Ciliated_NOR
5-Ciliated_SSC
6-CD8T Cell_NOR
6-CD8 T Cell_SSC
7-Dendritic Cell_NOR
7-Dendritic Cell_SSC
8-AT2_NOR
8-AT2_SSC
9-Myofibroblast_NOR
9-Myofibroblast_SSC
10-NK Cell_NOR
10-NK Cell_SSC
11-Capillary...
11-Capillary...
12-Club_NOR
12-Club_SSC
13-Mast Cell_NOR
13-Mast Cell_SSC
14-Proliferating...
14-Proliferating...
15-Pericyte SMC_NOR
15-Pericyte SMC_SSC
16-Goblet_NOR
16-Goblet_SSC
17-Basal_NOR
17-Basal_SSC
18-Lymphatic...
18-Lymphatic...
19-Adventitial...
19-Adventitial...
20-Arterial...
20-Arterial Endothelial_SSC
21-AT1_NOR
21-AT1_SSC
22-B Cell_NOR
22-B Cell_SSC
MT-CO2
250
200
150
100
50
0
0-FABP4 Macrophage_NOR
0-FABP4 Macrophage_SSC
1-SPP1 Macrophage_NOR
1-SPP1 Macrophage_SSC
2-CD14 Monocyte_NOR
2-CD14 Monocyte_SSC
3-Venous Endothelial_NOR
3-Venous Endothelial_SSC
4-CD4T Cell T Reg_NOR
4-CD4 T Cell T Reg_SSC
5-Ciliated_NOR
5-Ciliated_SSC
6-CD8T Cell_NOR
6-CD8 T Cell_SSC
7-Dendritic Cell_NOR
7-Dendritic Cell_SSC
8-AT2_NOR
8-AT2_SSC
9-Myofibroblast_NOR
9-Myofibroblast_SSC
10-NK Cell_NOR
10-NK Cell_SSC
11-Capillary...
11-Capillary...
12-Club_NOR
12-Club_SSC
13-Mast Cell_NOR
13-Mast Cell_SSC
14-Proliferating...
14-Proliferating...
15-Pericyte SMC_NOR
15-Pericyte SMC_SSC
16-Goblet_NOR
16-Goblet_SSC
17-Basal_NOR
17-Basal_SSC
18-Lymphatic...
18-Lymphatic...
19-Adventitial...
19-Adventitial...
20-Arterial...
20-Arterial Endothelial_SSC
21-AT1_NOR
21-AT1_SSC
22-B Cell_NOR
22-B Cell_SSC
CHI3L2
4
3.5
3
2.5
2
1.5
1
0.5
0
0-FABP4 Macrophage_NOR
0-FABP4 Macrophage_SSC
1-SPP1 Macrophage_NOR
1-SPP1 Macrophage_SSC
2-CD14 Monocyte_NOR
2-CD14 Monocyte_SSC
3-Venous Endothelial_NOR
3-Venous Endothelial_SSC
4-CD4T Cell T Reg_NOR
4-CD4 T Cell T Reg_SSC
5-Ciliated_NOR
5-Ciliated_SSC
6-CD8T Cell_NOR
6-CD8 T Cell_SSC
7-Dendritic Cell_NOR
7-Dendritic Cell_SSC
8-AT2_NOR
8-AT2_SSC
9-Myofibroblast_NOR
9-Myofibroblast_SSC
10-NK Cell_NOR
10-NK Cell_SSC
11-Capillary...
11-Capillary...
12-Club_NOR
12-Club_SSC
13-Mast Cell_NOR
13-Mast Cell_SSC
14-Proliferating...
14-Proliferating...
15-Pericyte SMC_NOR
15-Pericyte SMC_SSC
16-Goblet_NOR
16-Goblet_SSC
17-Basal_NOR
17-Basal_SSC
18-Lymphatic...
18-Lymphatic...
19-Adventitial...
19-Adventitial...
20-Arterial...
20-Arterial Endothelial_SSC
21-AT1_NOR
21-AT1_SSC
22-B Cell_NOR
22-B Cell_SSC

Figure S17
